# Metabolic profiles outperform the microbiota in assessing the response of vaginal microenvironments to the changed state of HPV infection

**DOI:** 10.1038/s41522-024-00500-0

**Published:** 2024-03-20

**Authors:** Wenkui Dai, Hui Du, Qian Zhou, Sumei Li, Yinan Wang, Jun Hou, Chunlei Guo, Qing Yang, Changzhong Li, Shouxia Xie, Shuai Cheng Li, Ruifang Wu

**Affiliations:** 1 Department of Obstetrics and Gynecology, Peking University Shenzhen Hospital, Shenzhen, China; 2 Institute of Obstetrics and Gynecology, Shenzhen PKU-HKUST Medical Center, Shenzhen, China; 3 Shenzhen Key Laboratory on Technology for Early Diagnosis of Major Gynecologic Diseases, Shenzhen, China; 4 Department of Computer Science, City University of Hong Kong, Hong Kong, China; 5 Department of Pharmacology, Shenzhen People’s Hospital (The Second Clinical Medical College, Jinan University, The First Affiliated Hospital, Southern University of Science and Technology), Shenzhen, China

**Keywords:** Health care, Microbial communities

## Abstract

There is a deficiency in population-based studies investigating the impact of HPV infection on vaginal microenvironment, which influences the risk of persistent HPV infection. This prospective study aimed to unravel the dynamics of vaginal microbiota (VM) and vaginal metabolome in reaction to the changed state of HPV infection. Our results propose that the vaginal metabolome may be a superior indicator to VM when assessing the impact of altered HPV state on the vaginal microenvironment.

The interaction between the human papillomavirus (HPV), host immune and vaginal microenvironment impacts the risk of persistent HPV infection and progression of cervical intraepithelial neoplasia (CIN)^1^. There is accumulated population-based evidence for HPV-modulated host responses and the impact of the vaginal microenvironment on the persistent risk of HPV infection^2–5^. In contrast, the impact of HPV on the vaginal microenvironment remains to be understood due to the unculturable nature of HPV. A recent study applied the animal model to demonstrate the downregulation of HPV oncoproteins on several innate peptides, that can be utilized as nutrient resources by vaginal *Lactobacillus* species in vitro^6^. However, population-based evidence is demanding due to the complexity of the human vaginal microenvironment. Surgical treatment is commonly provided to women with precancerous CIN, which eliminates persistent HPV infection in the vaginal microenvironment. Therefore, we can understand the impact of the HPV on vaginal microenvironment by analyzing differences between pre- and post-treatment.

This study aimed to unravel dynamic changes of both vaginal microbiota (VM) and vaginal metabolome in response to the surgical removal of high-grade CIN. Thus, we conducted a six-month follow-up investigation for 73 women who received surgery due to high-grade CIN (Fig. 1). Then dynamic changes of VM and vaginal metabolome were analyzed for 65 women, who experienced no vaginal infections and no re-infection of HPV after therapies (Fig. 1, Supplementary Table 1).

**Fig. 1:**
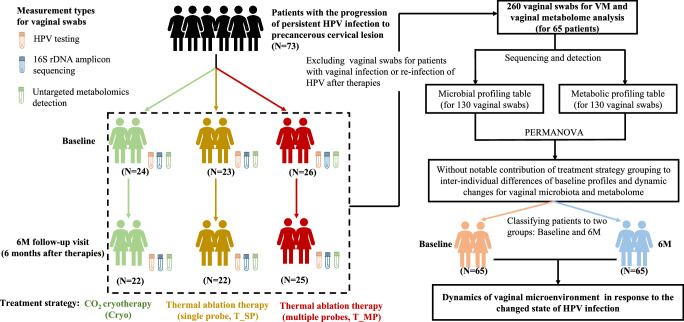
Workflow of this study. PERMANOVA indicated no statistically notable differences in VM and vaginal metabolome between Cryo, T_SP, and T_MP group. Therefore, all microbial samples were classified into two groups (baseline and 6 M), which were analyzed to understand the dynamics of both the VM and the vaginal metabolome.

Given three different surgical strategies, we applied the permutational multivariate analysis of variance (PERMANOVA) to assess if therapy strategy contributed to inter-individual variation for both VM and vaginal metabolome. We found no significant contribution of surgery strategy to microbial and metabolic profiles at baseline, as well as no significant separation between baseline and follow-up samples according to microbial profiles (Supplementary Fig. 1A, Supplementary Table 2). In addition, the dynamics of VM and vaginal metabolome were not impacted by therapy strategy (Supplementary Fig. 1B, C, Supplementary Table 2). We observed no significant contribution of other factors to baseline and follow-up microbial/metabolic profiles as well as their dynamics (Supplementary Fig. 1B, C, Supplementary Table 2). Thus, collected samples were divided into baseline and follow-up groups for subsequent analysis (Fig. 1).

We observed a partial but notable separation between the baseline and follow-up samples, based on the 389 metabolites generated of known identity (Fig. 2A), agreeing previous studies on differences of dynamics between microbial structure and transcriptional output^7,8^. These findings are partially explained by spatiotemporal characteristics of functional profiles for microbial communities and strains in response to environmental changes^9–11^. Further, the weighted correlation network analysis bins the metabolites into 11 co-abundance modules (MetaG) via WGCNA (v.1.69). Four and one modules were notably depleted or enriched at 6 M follow-up visits, respectively (Fig. 2B, Supplementary Fig. 2). The 6M-enriched module (MetaG10) comprised 27 metabolites, mainly glycerophospholipids and glycerolipids (Fig. 2C, Supplementary Table 3). In contrast, metabolites in 6M-depleted MetaG1 and MetaG5 were mainly organic nitrogen compounds, as well as organic acids and derivatives, including amino acids, peptides, and analogs (Fig. 2C, Supplementary Table 3). However, functional studies should be performed to understand the role of these metabolites in HPV infection.

**Fig. 2:**
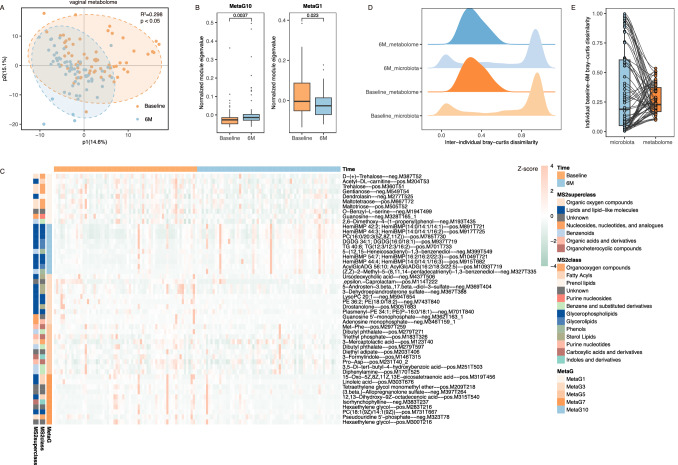
Characterization of vaginal metabolome dynamics. **A** Partial least squares discriminant analysis (PLS-DA) shows a partial but notable separation between baseline and follow-up samples for the vaginal metabolome. Significance was determined using permutational multivariate analysis of variance. **B** The resultant metabolite abundance matrix was then subjected to co-abundance clustering using WGCNA. Box plots indicate the normalized module eigenvalue of MetaG10 (*p* = 0.0037) and MetaG1 (*p* = 0.023). The central line indicates the median. The lower and upper hinges indicate the first and third quartiles. Significance was determined using the Wilcoxon signed rank test. **C** Leave-one-out analysis was conducted to determine the contribution of 389 identified metabolites in differentiating baseline and follow-up groups. The top ten essential metabolites in MetaG10, 1, 3, 5, and 7 are shown for display purposes. For 50 displayed metabolites, the normalized abundances (z-score normalization of the log2 transformed intensity) are shown in the heatmap. **D** The distribution of bray-curtis distance for VM and vaginal metabolome at baseline and 6 M follow-up. **E** For the 65 patients included, the intra-individual distance was calculated based on the VM and the vaginal metabolome, respectively. The central line indicates the median. The lower and upper hinges indicate the first and third quartiles.

Subsequent analysis identified a notable association of the above-mentioned metabolic modules with several low-abundance VM components (Supplementary Fig. 3), which is partially explained by inconsistent dynamics between the level of vaginal microbes and their functional outputs^7^. For example, low-abundance *Streptococcus*, which level increased after surgeries, was positively associated with 6M-accumulated MetaG10, and had a negative association with 6M-depleted MetaG3, as well as MetaG7 (Supplementary Figs. 3 and 4). In addition, 6M-depleted MetaG1 and MetaG7 positively correlated with *Prevotella*, whose level increased in persistent HPV infection (Supplementary Figs. 3 and 4)^12^. And these two modules are negatively associated with *Lactobacillus*, which is crucial for vaginal health and commonly dominates the VM of healthy women (Supplementary Figs. 3 and 4)^13,14^. Nevertheless, prior studies observed the association of other low-abundance microbes like *Sneathia, Bacteroides*, and *Halomonas* with the status of HPV infection and CIN^15–17^. Given the functional redundancy of microbial strains^18^, similar functional results can be found in various vaginal microbes, which partially explains the heterogeneous findings on the association of low-abundance VM components with HPV infection.

Moreover, we identified a lower inter-individual heterogeneity of the vaginal metabolome than of the VM, partly due to above-mentioned functional redundancy^18^. Being assessed by bray-curtis dissimilarity, vaginal metabolome displays lower inter-individual variations than VM at both baseline and follow-up visits (Fig. 2D). According to VM, the inter-individual bray-curtis for most of the microbial samples was larger than 0.9. The inter-individual bray-curtis of microbial samples was 0.3 based on the vaginal metabolome (Fig. 2D). For dynamic changes, metabolic profiles also had lower inter-individual dissimilarity for time-related intra-individual distance when compared to microbial profiles (Fig. 2E). Taking the subject P23 for example, the bray-curtis distance of microbial samples was 0.973 and 0.196 based on VM and vaginal metabolome, respectively.

Besides high sensitivity to environmental changes and low inter-individual variations, the vaginal metabolome represents the products of HPV-host-VM interactions in the vaginal microenvironment. Therefore, the vaginal metabolome should outperform VM in assessing the response of the vaginal microenvironment to the change in the state of HPV infection. Similarly, several studies suggested that functional omics data outperformed microbial structures in assessing the response to environmental changes^8,19,20^.

Despite above-mentioned findings, several limitations exist for this study. First, the small sample size limited the deep understanding of VM and vaginal metabolome dynamics. For example, only three patients were reinfected with HPV. Thus, we could not analyze the differences in microenvironment dynamics between HPV-negative and re-infected women after surgeries. Second, no cytology was performed at the follow-up visit. However, this study aimed to assess the response to the changed state of HPV infection. Therefore, the lack of cytological results should not negatively affect our findings. Third, 16S rDNA amplicon sequencing hindered the species-level analysis of VM and limited the correlation analysis with metabolomic data.

This prospective study indicated notable changes of vaginal metabolome after eliminating high-grade CIN. This study should provide crucial population-based evidence for understanding the impact of HPV on the vaginal microenvironment.

## Methods

### Ethics approval

This study was approved by the Ethics Committee of Peking University Shenzhen Hospital (registration number: 2021-006). All participants were fully informed and then provided signed consent.

### Patient recruitment and sample collection

Attenders were recruited by interview and the metadata was recorded by the clinician. HPV-positive women were included following the inclusion criteria: receiving CO_2_ cryotherapy or thermal ablation (single or multiple probes) for high-grade cervical intraepithelial neoplasia; older than 18 years and no menopause; no history of cervical ablation or resection surgery or hysterectomy or pelvic radiotherapy; no douching and vaginal medications within seven days; no antibiotics exposures with one month; no hormone replacement therapy or GnRH-a within three months; no autoimmune diseases or HIV infection; having negative results for H2O2, leukocyte esterase, neuraminidase, b-glucuronidase or acetylaminoglucosidase (detected by the bPR2014A platform, Jiangsu Bioperfectus Technologies Co., Ltd.) to exclude common genital infections. Then vaginal swabs was sampled ≥3 days after menstruation and collected at posterior fornix by a clinician before and six months after therapy. Vaginal swabs was collected at posterior fornix by a clinician before and six months after therapy. Collected swabs were preserved in a 2 ml sterile tube and stored at −80 centigrade within 30 min after collection. Vaginal swabs from 65 women were included for further analysis according to the criteria: without re-infection of HPV; having negative results for H2O2, leukocyte esterase, neuraminidase, b-glucuronidase, or acetylamino glucosidase at follow-up visit. Vaginal swabs from 65 women were included for further analysis according to the criteria: without re-infection of HPV; having negative results for H_2_O_2_, leukocyte esterase, neuraminidase, b-glucuronidase, or acetylamino glucosidase at follow-up visit.

### Microbial DNA extraction and 16S rDNA amplicon sequencing

Microbial DNA of vaginal swabs was extracted one month after collection by Dneasy PowerSoil Pro Kit (Qiagen, German) and then stored at −80 centigrade. DNA concentration and purity were estimated by 1% agarose gels on Agilent5400 (Agilent Technologies, Inc., Santa Clara, USA). Then we amplified 16S rDNA V4-V5 hypervariable regions (515-FR: GTGCCAGCMG CCGCGGTAA, 926-RR: CCGTCAATTCMTTTRAGTTT) of 16S rRNA gene and determined the quality of PCR products (Qubit, Thermo Fisher Scientific, Singapore). Subsequently, DNA libraries were sequenced by 250 bp read length based on Illumina NovaSeq platform (Illumina, San Diego, CA, United States). Sequencing data was processed and analzyed via QIIME 2 to output the profiling table of VM for subsequent analysis.

### Metabolomics analysis

Untargeted metabolomics characterization was performed via LC-MS based on Thermo Fisher Scientific (Ottawa, United States). Raw metabolome data were then converted into mzXML format and ion features were extracted using Progenesis QI (v.2.2). Then the ions were filtered as they fulfilled one of the following criteria: missing in more than 50% of the quality control samples or more than 80% of the research samples; having a relative standard deviation >30%. Metabolite annotation was conducted via searching HMDB (v.5.0) and KEGG (v.96.0) databases. The resultant metabolite abundance matrix was used for subsequent analysis.

### Statistics analysis

The effect of different factors on VM/vaginal metabolome structure was assessed by the PERMANOVA test with 9999 permutations (package “vegan” in R). To quantify the percentage of variance explained by various factors on VM/vaginal metabolome dynamics, vectors of person-specific changes were calculated for each open taxonomic unit (OTU) and metabolite per individual as follows: Log2(OTU_6M_/OTU_baseline_) and Log2(metabolite_6M_/metabolite_baseline_). Then we applied PERMANOVA to assess the explained variance of each factor based on these vectors. Wilcoxon signed-rank test was applied to analyze inter-group differences, and the *p*-value was adjusted by the Benjamini & Hochberg method. WGCNA (v.1.69) was applied to bin the metabolites to modules. Analysis results were visualized using R software (v.4.05).

### Reporting summary

Further information on research design is available in the Nature Research Reporting Summary linked to this article.

### Supplementary information

Supplementary File

nr-reporting-summary

## Data Availability

The data of 16S rDNA amplicon sequencing was submitted to CNGB Sequence Archive (CNSA) under Project No. CNP0004255 and CNP0002763. The annotated metabolomes datasets are available at CNSA (https://db.cngb.org/search/metabolize/METM0000175/). Authors declare that all other data supporting the findings of the study are available from the corresponding authors on reasonable request.
