# Supplementary material for: Metabolic profiles outperform the microbiota in assessing the response of vaginal microenvironments to the changed state of HPV infection
Source: NPJ Biofilms Microbiomes. 2024 Mar 20;10:26. doi: 10.1038/s41522-024-00500-0 (PMC10954630; doi:10.1038/s41522-024-00500-0)
Supplement: Supplementary file 1 — Supplementary File [file 41522_2024_500_MOESM1_ESM.pdf]

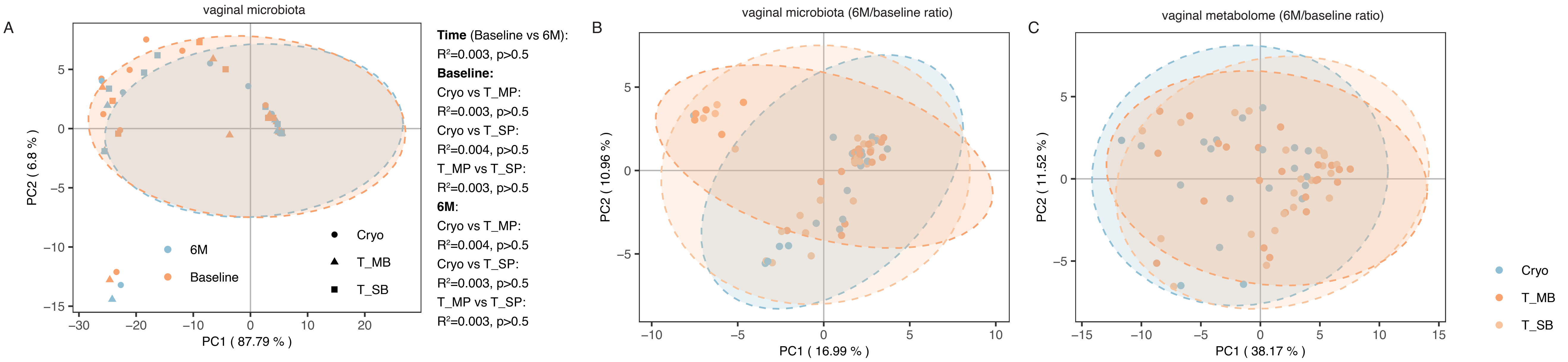

Supplementary Figure 1. The explained variance of several factors on microbial and metabolic profiles as well as dynamics. A. Principal coordinates analysis of both vaginal microbiota (VM) and vaginal metabolome. There was no significant separation of microbial samples between baseline and six months post-therapy (6M) .Therapy strategies had insignificant contribution to dynamics of VM and vaginal metabolome. **B.** First vectors of person-specific open taxonomic unit (OTU) changes were calculated for each OTU per individual as follows: Log_2 (OTU6M/OTUBaseline). Then PERMANOVA was applied to analyze explained variance of several factors on VM dynamics. **C.** First vectors of person-specific metabolite changes were calculated for each metabolite per individual as follows: Log_2 (metabolite6M/metaboliteBaseline). Then PERMANOVA was applied to analyze explained variance of several factors on dynamic changes of vaginal metabolome. R^2 represents explained variance.

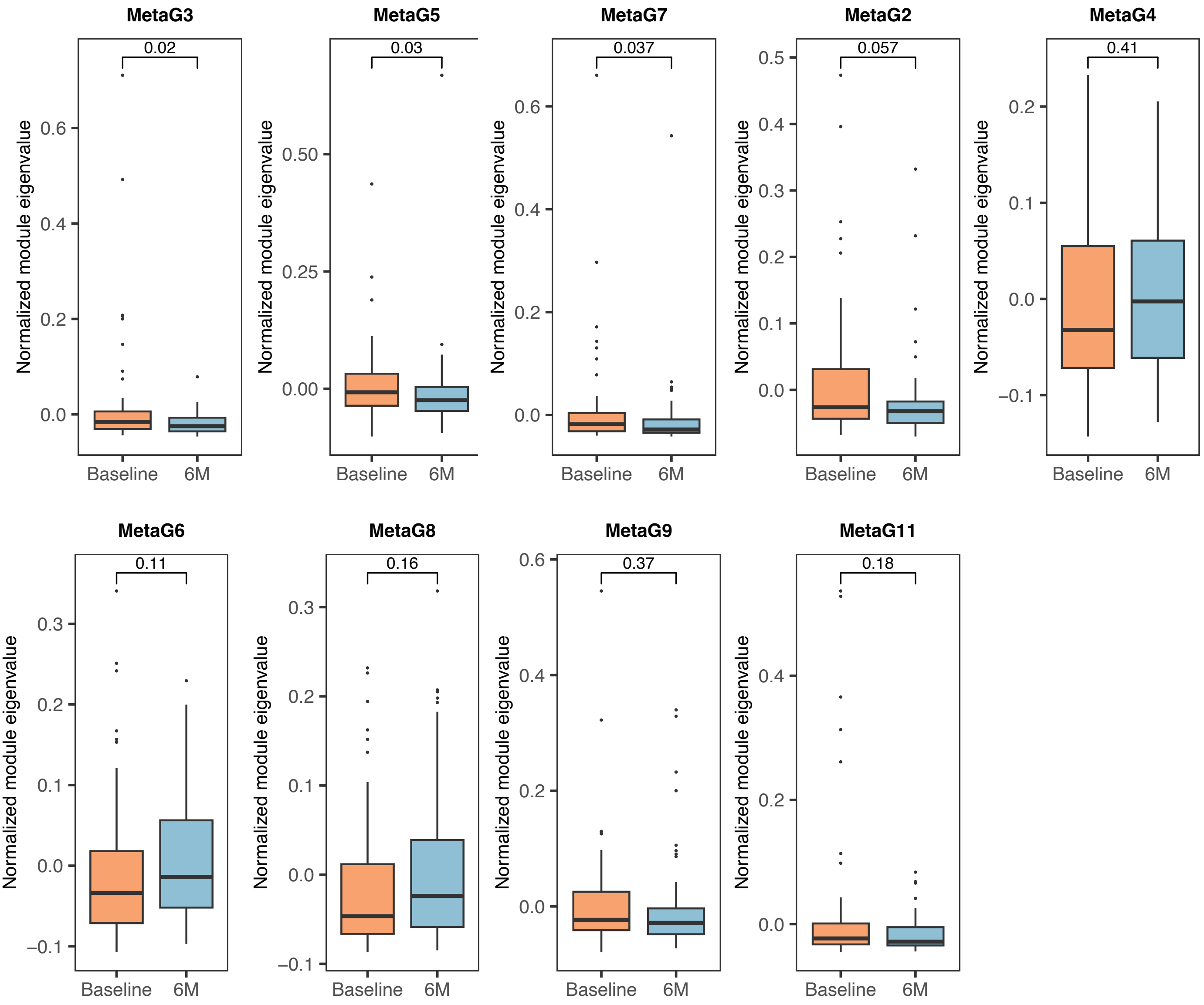

Supplementary Figure 2. Dynamic changes of metabolite modules. Box plots indicate the normalized module eigenvalue of metabolite modules. The central line indicates the median. The lower and upper hinges indicate the first and third quartiles. Significance was determined using the Wilcoxon signed-rank test.

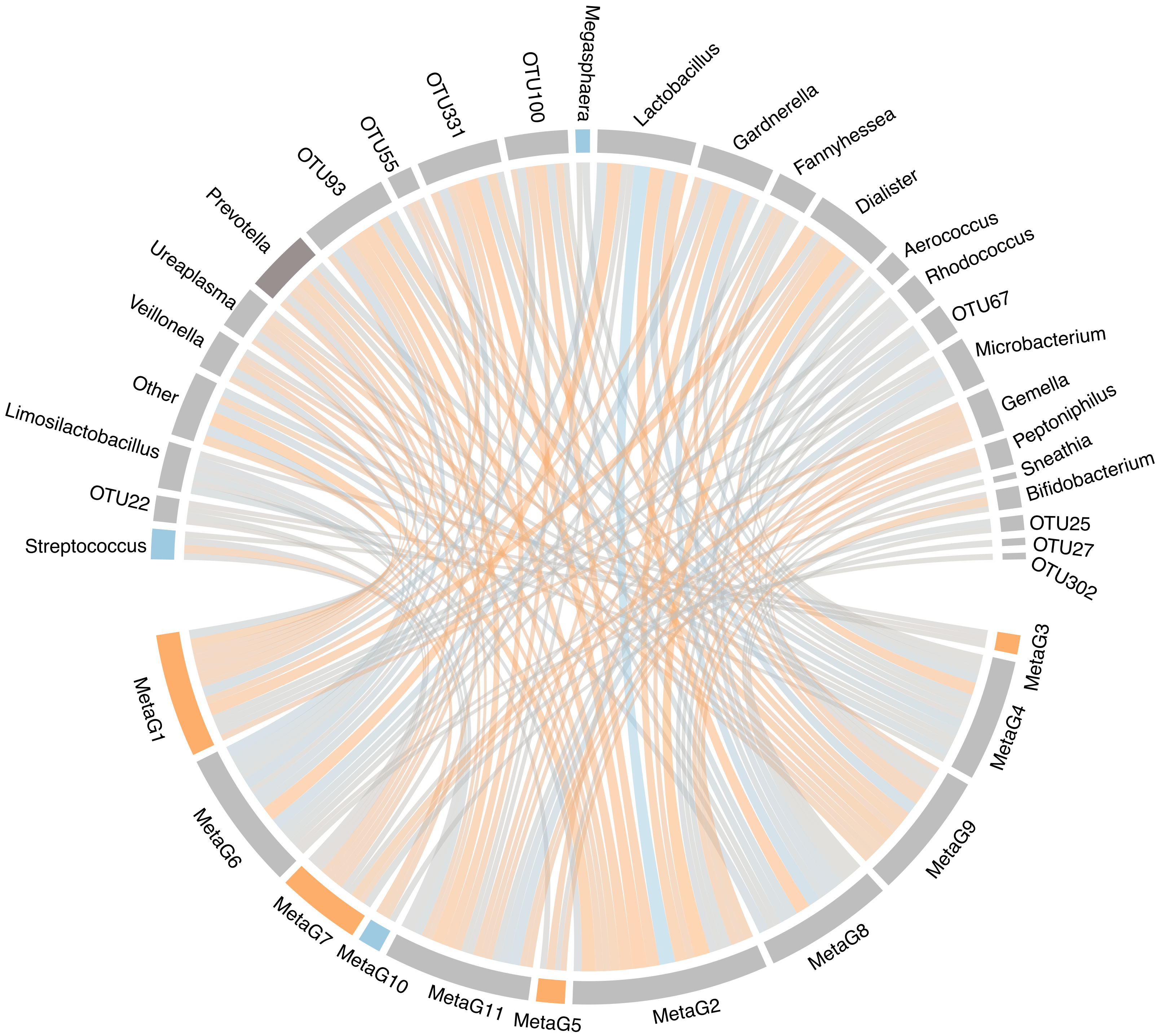

Supplementary Figure 3. Association of metabolite modules with VM. The spearman correlation was calculated and the association significance was adjusted by false discovery rate. Correlations with > 0.1 coefficient and adjusted p value < 0.05 are displayed: orange and blue lines represent positive and negative correlation, respectively. The line width is proportional to the absolute value of correlation coefficient.

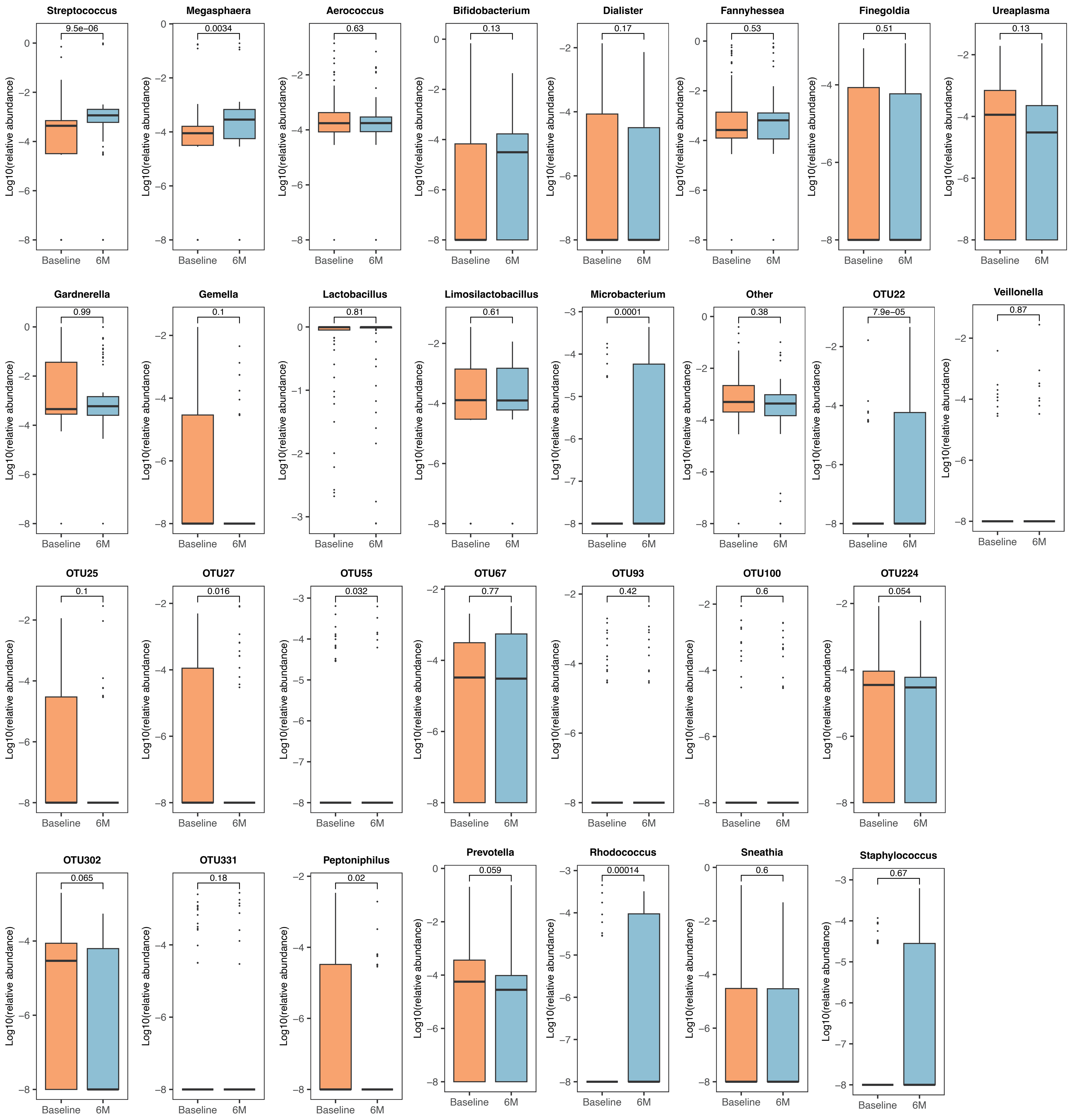

Supplementary Figure 4. Genus-level dynamic changes of VM. Box plots indicate the $\log_{10}$ value of relative abundance. The central line indicates the median. The lower and upper hinges indicate the first and third quartiles. Significance was determined using the Wilcoxon signed-rank test.

Supplementary Table 1. Information of included 65 patients

Therapy strategy	CO_2 cryotherapy (Cryo, N=20)	Thermal ablation (single probe, T_SP, N=22)	Thermal ablation (multiple probes, T_MP, N=23)
**Age (Average: min-max)**	27.9(21-40)	30.4(19-37)	28.9(23-38)
**HPV infection**			
HPV16+	45%(9/20)	41%(9/22)	52%(12/23)
HPV18+	5%(1/20)	1%(2/22)	13%(3/23)
Other 12 HPV genotypes+	65%(13/20)	77%(17/22)	52%(12/23)
**CIN grade**			
CIN2	90%(18/20)	86%(19/22)	70%(16/23)
CIN3	10%(2/20)	14%(3/22)	30%(7/23)
**Vaccination**			
4-valent	5%(1/20)	18%(4/22)	13%(3/23)
9-valent	10%(2/20)	23%(5/22)	17%(4/23)
**Smoking**	25%(5/20)	5%(1/22)	4%(1/23)
**No. of sex partner (Average: min-max)**	3(1-8)	2.9(1-10)	3(1-10)
**Age with first sex activity (Average: min-max)**	20(17-26)	20.8(15-30)	20(17-25)
**Contraception**	95%(19/20)	86%(19/22)	78%(18/23)
**Gestation**	50%(10/20) (Frequency: 1-5)	45%(10/22) (Frequency:1-3)	57%(13/23) (Frequency: 1-5)
**Abortion**	40%(8/20) (Frequency:1-3)	41%(9/22) (Frequency:1-2)	48%(11/23) (Frequency: 1-2)

Supplementary Table 2. PERMANOVA to assess the contribution of several factors to microbial and metabolic profiles as well as dynamics

		Microbiota			Metabolome		
		Baseline	6M	6M/Baseline	Baseline	6M	6M/Baseline
Age	R^2	0.013	0.005	0.028	0.027	0.023	0.014
	P	0.459	0.853	0.211	0.112	0.180	0.585
HPV16+	R^2	0.003	0.025	0.014	0.013	0.005	0.017
	P	0.965	0.162	0.574	0.510	0.929	0.327
HPV18+	R^2	0.008	0.031	0.012	0.040	0.018	0.044
	P	0.698	0.063	0.793	0.126	0.295	0.124
HPV_other+	R^2	0.017	0.059	0.016	0.015	0.016	0.017
	P	0.347	0.112	0.392	0.432	0.348	0.347
CIN grade	R^2	0.048	0.061	0.047	0.159	0.090	0.062
	P	0.409	0.215	0.433	0.228	0.122	0.197
Vaccination	R^2	0.007	0.013	0.032	0.016	0.014	0.024
	P	0.994	0.889	0.321	0.900	0.941	0.985
Smoking	R^2	0.008	0.013	0.014	0.017	0.009	0.014
	P	0.716	0.456	0.599	0.327	0.748	0.626
No. of sex partner	R^2	0.009	0.032	0.011	0.033	0.007	0.023
	P	0.651	0.098	0.752	0.174	0.851	0.129
Age of first sex	R^2	0.008	0.008	0.013	0.015	0.019	0.018
	P	0.694	0.674	0.685	0.407	0.257	0.309
Contraception	R^2	0.015	0.007	0.016	0.005	0.027	0.003
	P	0.396	0.745	0.416	0.952	0.117	0.984
Gestation	R^2	0.009	0.023	0.032	0.046	0.025	0.032
	P	0.680	0.199	0.204	0.116	0.143	0.183
Gestation frequency	R^2	0.017	0.046	0.019	0.032	0.052	0.020
	P	0.343	0.134	0.174	0.171	0.211	0.244
Abortion	R^2	0.016	0.029	0.025	0.018	0.022	0.016
	P	0.366	0.123	0.134	0.292	0.185	0.370
Abortion frequency	R^2	0.020	0.034	0.020	0.025	0.037	0.018
	P	0.247	0.188	0.149	0.160	0.148	0.292
Therapy strategy	R^2	0.046	0.033	0.03	0.032	0.019	0.03
	P	0.158	0.375	0.62	0.408	0.822	0.65

PERMANOVA: permutational multivariate analysis of variance. 6M: six months post-therapy. HPV: human papillomavirus. CIN: cervical intraepithelial neoplasia.

Supplementary Table 3

ID	Importance	MetaG	Metabolite	Superclass	Class	Subclass
pos.M891T721	1.179895834	MetaG10	HemiBMP 42:2; HemiBMP(14:0/14:1/14:1)	Lipids and lipid-like molecules	Glycerophospholipids	NA
pos.M917T725	1.047648463	MetaG10	HemiBMP 44:3; HemiBMP(14:0/14:1/16:2)	Lipids and lipid-like molecules	Glycerophospholipids	NA
pos.M785T730	0.995331451	MetaG10	PC(16:0/20:3(5Z,8Z,11Z))	Lipids and lipid-like molecules	Glycerophospholipids	Glycerophosphocholines
pos.M759T726	0.882134504	MetaG9	PC(16:0/18:2(9Z,12Z))	Lipids and lipid-like molecules	Glycerophospholipids	Glycerophosphocholines
pos.M937T719	0.846028887	MetaG10	DGDG 34:1; DGDG(16:0/18:1)	Lipids and lipid-like molecules	Glycerolipids	NA
pos.M303T581	0.804551943	MetaG2	Linoleic acid	Lipids and lipid-like molecules	Fatty Acyls	Lineolic acids and derivatives
pos.M701T733	0.665568117	MetaG10	TG 40:8; TG(12:3/12:3/16:2)	Lipids and lipid-like molecules	Glycerolipids	NA
neg.M437T506	0.630107041	MetaG3	Ursodeoxycholic acid	Lipids and lipid-like molecules	Sterol Lipids	Bile acids, alcohols and derivatives
pos.M319T456	0.582819129	MetaG7	15-Oxo-5Z,8Z,11Z,13E-eicosatetraenoic acid	Lipids and lipid-like molecules	Fatty Acyls	Fatty acids and conjugates
pos.M303T676	0.569393332	MetaG7	Linoleic acid	Lipids and lipid-like molecules	Fatty Acyls	Lineolic acids and derivatives
pos.M114T222	0.568743432	MetaG3	.epsilon.-Caprolactam	Unknown	Unknown	NA
neg.M399T549	0.500923037	MetaG10	5-(12,15-Heneicosadienyl)-1,3-benzenediol	Benzenoids	Phenols	Benzenediols
pos.M1049T721	0.492178446	MetaG10	HemiBMP 54:7; HemiBMP(16:2/16:2/22:3)	Lipids and lipid-like molecules	Glycerophospholipids	NA
pos.M124T78	0.447806416	MetaG2	Picolinic acid	Organoheterocyclic compounds	Pyridines and derivatives	Pyridinecarboxylic acids and derivatives
neg.M369T404	0.444300086	MetaG3	5-Androsten-3.beta.,17.beta.-diol-3-sulfate	Lipids and lipid-like molecules	Sterol Lipids	NA
pos.M915T682	0.440074193	MetaG10	HemiBMP 44:4; HemiBMP(14:0/14:1/16:3)	Lipids and lipid-like molecules	Glycerophospholipids	NA
pos.M1093T719	0.42385177	MetaG10	AcylGlcADG 56:10; AcylGlcADG(16:2/18:3/22:5)	Lipids and lipid-like molecules	Glycerolipids	NA
pos.M313T283	0.40287035	MetaG4	Phenylalanylphenylalanine	Organic acids and derivatives	Carboxylic acids and derivatives	Amino acids, peptides, and analogues
pos.M623T545	0.387084633	MetaG9	(S,S)-(+)-Tetrandrine	ıans, neolignans and related compoı	Unknown	NA
pos.M214T475	0.37836808	MetaG6	Tetradecylamine	Unknown	Unknown	NA
pos.M209T218	0.376441798	MetaG7	Tetraethylene glycol monomethyl ether	Unknown	Unknown	NA
neg.M572T584	0.36519738	MetaG11	LysoPS 22:4; LysoPS 22:4	Lipids and lipid-like molecules	Glycerophospholipids	NA
pos.M286T500	0.354439393	MetaG4	C17_Sphingosine	Unknown	Unknown	NA
neg.M327T335	0.342288371	MetaG10	-Methyl-5-(8,11,14-pentadecatrienyl)-1,3-benz	Benzenoids	Phenols	Benzenediols
neg.M611T155	0.339863751	MetaG11	L-Glutathione (oxidized form)	Organic acids and derivatives	Carboximidic acids and derivatives	NA
pos.M297T259	0.336825266	MetaG5	Met-Phe	Organic acids and derivatives	Carboxylic acids and derivatives	Amino acids, peptides, and analogues
neg.M397T264	0.334524934	MetaG7	(3.beta.)-Allopregnanolone sulfate	Unknown	Unknown	NA
pos.M300T526	0.319304748	MetaG4	D-erythro-N-stearoylsphingosine	Organic nitrogen compounds	Organonitrogen compounds	Amines

pos.M279T271	0.315660294	MetaG5	Dibutyl phthalate	Benzenoids	Benzene and substituted derivatives	Benzoic acids and derivatives
pos.M770T812_1	0.31279813	MetaG9	TG 45:9; TG(15:3/15:3/15:3)	Lipids and lipid-like molecules	Glycerolipids	NA
neg.M367T388	0.30722184	MetaG3	3-Dehydroepiandrosterone sulfate	Lipids and lipid-like molecules	Sterol Lipids	Sulfated steroids
neg.M594T654	0.307180718	MetaG3	LysoPC 20:1	Lipids and lipid-like molecules	Glycerophospholipids	NA
neg.M743T840	0.305349669	MetaG3	PE 36:2; PE(18:0/18:2)	Lipids and lipid-like molecules	Glycerophospholipids	Glycerophosphoethanolamines
pos.M233T147_1	0.300765674	MetaG4	Isoleucyl-Threonine	Organic acids and derivatives	Carboxylic acids and derivatives	Amino acids, peptides, and analogues
pos.M315T540	0.291525397	MetaG7	12,13-Dihydroxy-9Z-octadecenoic acid	Lipids and lipid-like molecules	Fatty Acyls	Fatty acids and conjugates
neg.M327T319	0.285685856	MetaG10	-Methyl-5-(8,11,14-pentadecatrienyl)-1,3-benz	Benzenoids	Phenols	Benzenediols
neg.M387T52	0.280488886	MetaG1	D-(+)-Trehalose	Organic oxygen compounds	Organooxygen compounds	arbohydrates and carbohydrate conjugate
neg.M558T75	0.276522895	MetaG9	Phosphoribosyl-AMP	ucleosides, nucleotides, and analogu	Purine nucleotides	Purine ribonucleotides
neg.M383T237	0.275018086	MetaG7	Isorhynchophylline	Organoheterocyclic compounds	Indoles and derivatives	NA
pos.M305T683	0.273718605	MetaG3	Drostanolone	Lipids and lipid-like molecules	Sterol Lipids	Androstane steroids
pos.M204T53	0.271304389	MetaG1	Acetyl-DL-carnitine	Lipids and lipid-like molecules	Fatty Acyls	Fatty acid esters
pos.M135T39	0.269272042	MetaG4	Protoanemonin	Organoheterocyclic compounds	Dihydrofurans	Furanones
pos.M283T216	0.268645508	MetaG7	Hexaethylene glycol	Unknown	Unknown	NA
pos.M716T643	0.260647101	MetaG10	2,2',3,4,4',5',6-Heptabromodiphenyl ether	Benzenoids	Benzene and substituted derivatives	Diphenylethers
pos.M731T667	0.258847965	MetaG7	PC(18:1(9Z)/14:1(9Z))	Lipids and lipid-like molecules	Glycerophospholipids	Glycerophosphocholines
neg.M214T48	0.25877759	MetaG4	N-Methylethanolaminium phosphate	Organic acids and derivatives	rganic phosphoric acids and derivative	Phosphate esters
pos.M360T51	0.251406164	MetaG1	Trehalose	Organic oxygen compounds	Organooxygen compounds	arbohydrates and carbohydrate conjugate
neg.M131T265	0.249923959	MetaG2	Tetrahydrofuran	Organoheterocyclic compounds	Oxolanes	NA
pos.M783T687	0.248649351	MetaG10	PC(18:2(9Z,12Z)/18:2(9Z,12Z))	Lipids and lipid-like molecules	Glycerophospholipids	Glycerophosphocholines
pos.M274T162_1	0.24507457	MetaG6	Val-Gly-Val	Organic acids and derivatives	Carboxylic acids and derivatives	NA
neg.M323T78	0.237732759	MetaG7	Pseudouridine 5'-phosphate	Organic oxygen compounds	Organooxygen compounds	arbohydrates and carbohydrate conjugate
neg.M544T576	0.236574812	MetaG11	LysoPS 20:4; LysoPS 20:4	Lipids and lipid-like molecules	Glycerophospholipids	NA
pos.M300T216	0.235552492	MetaG7	Hexaethylene glycol	Unknown	Unknown	NA
pos.M334T243	0.23436101	MetaG6	Glu-Trp	Organic acids and derivatives	Carboxylic acids and derivatives	Amino acids, peptides, and analogues
neg.M313T465	0.234104877	MetaG7	9,10-Dihydroxy-12Z-octadecenoic acid	Lipids and lipid-like molecules	Fatty Acyls	Fatty acids and conjugates
neg.M558T53	0.232634294	MetaG9	Phosphoribosyl-AMP	ucleosides, nucleotides, and analogu	Purine nucleotides	Purine ribonucleotides
pos.M183T326	0.230503762	MetaG5	Triethyl phosphate	Organic acids and derivatives	Carboxylic acids and derivatives	NA
pos.M146T211	0.227856718	MetaG4	L-Leucine, methyl ester	Organic acids and derivatives	Carboxylic acids and derivatives	NA

pos.M331T617	0.227844851	MetaG2	8Z,14Z-Eicosadienoic acid	Lipids and lipid-like molecules	Fatty Acyls	NA
neg.M701T840	0.227183277	MetaG3	Plasmenyl-PE 34:1; PE(P-16:0/18:1)	Lipids and lipid-like molecules	Glycerophospholipids	NA
neg.M362T163_1	0.226034771	MetaG3	Guanosine 5'-monophosphate	ucleosides, nucleotides, and analogu	Unknown	NA
pos.M260T160	0.225833489	MetaG6	Leu-Lys	Organic acids and derivatives	Carboxylic acids and derivatives	Amino acids, peptides, and analogues
neg.M549T54	0.224348995	MetaG1	Gentianose	Organic oxygen compounds	Organooxygen compounds	arbohydrates and carbohydrate conjugate
pos.M414T601	0.219280848	MetaG4	Acylcarnitine 17:0	Lipids and lipid-like molecules	Fatty Acyls	Fatty acid esters
pos.M245T231	0.218122651	MetaG4	Isoleucyl-Leucine	Organic acids and derivatives	Carboxylic acids and derivatives	Amino acids, peptides, and analogues
pos.M305T595	0.216521408	MetaG2	Drostanolone	Lipids and lipid-like molecules	Sterol Lipids	Androstane steroids
pos.M295T574	0.215337863	MetaG2	9-Oxo-10(E),12(E)-octadecadienoic acid	Unknown	Unknown	NA
pos.M265T226	0.2126412	MetaG6	Phenylalanyl-Valine	Organic acids and derivatives	Carboxylic acids and derivatives	Amino acids, peptides, and analogues
pos.M319T590	0.212396414	MetaG2	12-Oxo-5Z,8Z,10E,14Z-eicosatetraenoic acid	Lipids and lipid-like molecules	Fatty Acyls	Fatty acids and conjugates
neg.M178T245	0.212391615	MetaG6	Cyclamate	Organic acids and derivatives	Sulfamic acid derivatives	Cyclamates
pos.M324T620	0.210971169	MetaG6	5Z,8Z,14Z-Eicosatrienoic acid	Lipids and lipid-like molecules	Fatty Acyls	NA
pos.M182T159	0.208792594	MetaG4	L-Tyrosine	Organic acids and derivatives	Carboxylic acids and derivatives	Amino acids, peptides, and analogues
neg.M604T78	0.206226762	MetaG9	Guanosine 5'-diphosphate-D-mannose	ucleosides, nucleotides, and analogu	Purine nucleotides	NA
pos.M919T778	0.204713892	MetaG9	HemiBMP 44:2; HemiBMP(12:0/16:1/16:1)	Lipids and lipid-like molecules	Glycerophospholipids	NA
neg.M427T246	0.204592026	MetaG7	Irbesartan	Benzenoids	Benzene and substituted derivatives	Biphenyls and derivatives
neg.M346T159_1	0.201505503	MetaG3	Adenosine monophosphate	ucleosides, nucleotides, and analogu	Purine nucleotides	Purine ribonucleotides
neg.M227T43	0.199021778	MetaG9	2,3-Dimercaptosuccinic acid	Lipids and lipid-like molecules	Fatty Acyls	Fatty acids and conjugates
pos.M144T52_2	0.198136678	MetaG7	Stachydrine	Organic acids and derivatives	Carboxylic acids and derivatives	Amino acids, peptides, and analogues
pos.M296T169	0.196482542	MetaG6	Norfluoxetine	Benzenoids	Benzene and substituted derivatives	Trifluoromethylbenzenes
neg.M503T53	0.192787819	MetaG6	1-Kestose	Organic oxygen compounds	Organooxygen compounds	arbohydrates and carbohydrate conjugate
pos.M753T567	0.192671707	MetaG10	PC(14:1(9Z)/20:4(5Z,8Z,11Z,14Z))	Lipids and lipid-like molecules	Glycerophospholipids	Glycerophosphocholines
neg.M789T841	0.191333349	MetaG3	PS 36:1; PS(18:0/18:1)	Lipids and lipid-like molecules	Glycerophospholipids	Glycerophosphoserines
pos.M269T114	0.190622646	MetaG6	Tyr-Ser	Organic acids and derivatives	Carboxylic acids and derivatives	Amino acids, peptides, and analogues
neg.M496T571	0.188813193	MetaG3	LysoPS 16:0; LysoPS 16:0	Lipids and lipid-like molecules	Glycerophospholipids	NA
neg.M421T238	0.188682264	MetaG4	Riboflavin	Organoheterocyclic compounds	Pteridines and derivatives	Alloxazines and isoalloxazines
neg.M476T839	0.187303808	MetaG3	LysoPE 18:2	Lipids and lipid-like molecules	Glycerophospholipids	Glycerophosphoethanolamines
neg.M619T530	0.185974264	MetaG2	LysoPI 20:4; LysoPI 20:4	Lipids and lipid-like molecules	Glycerophospholipids	NA
neg.M277T525	0.183885599	MetaG1	Dendrolasin	Lipids and lipid-like molecules	Prenol lipids	Monoterpenoids

pos.M352T658	0.183532569	MetaG7	Docosatrienoic acid	Lipids and lipid-like molecules	Fatty Acyls	Fatty acids and conjugates
pos.M123T40	0.182511841	MetaG5	3-Mercaptolactic acid	Organic oxygen compounds	Organooxygen compounds	Amino acids, peptides, and analogues
pos.M176T49	0.18224347	MetaG2	L-Citrulline	Organic acids and derivatives	Carboxylic acids and derivatives	Amino acids, peptides, and analogues
pos.M393T225	0.181215331	MetaG10	Racemoramide	Unknown	Unknown	NA
pos.M304T84	0.177349355	MetaG4	Glu-Arg	Organic acids and derivatives	Carboxylic acids and derivatives	Amino acids, peptides, and analogues
pos.M667T72	0.177299194	MetaG1	Maltotetraose	Organic oxygen compounds	Organooxygen compounds	arbohydrates and carbohydrate conjugate
pos.M294T213	0.176806581	MetaG6	Phenylalanyl-Gamma-glutamate	Organic acids and derivatives	Carboxylic acids and derivatives	Amino acids, peptides, and analogues
pos.M318T508	0.176243688	MetaG3	Phytosphingosine	Organic nitrogen compounds	Organonitrogen compounds	Amines
pos.M130T45	0.175794264	MetaG2	L-Pipecolic acid	Organic acids and derivatives	Carboxylic acids and derivatives	Amino acids, peptides, and analogues
pos.M505T52	0.172960381	MetaG1	Maltotriose	Organic oxygen compounds	Organooxygen compounds	arbohydrates and carbohydrate conjugate
pos.M153T38	0.172653346	MetaG4	cis-Acetylacrylate	Organic acids and derivatives	Keto acids and derivatives	Short-chain keto acids and derivatives
neg.M258T48	0.172546044	MetaG4	Iminoerythrose 4-phosphate	Organic acids and derivatives	rganic phosphoric acids and derivative	Phosphate esters
pos.M376T449	0.171727875	MetaG9	Linoleoylglycine	Unknown	Unknown	NA
pos.M377T241	0.171247356	MetaG4	(-)-Riboflavin	Organic oxygen compounds	Organooxygen compounds	Alcohols and polyols
pos.M268T151	0.170938288	MetaG6	Adenosine	ıcleosides, nucleotides, and analogu	Purine nucleosides	NA
pos.M162T53	0.170678934	MetaG4	2-Aminoadipic acid	Organic acids and derivatives	Carboxylic acids and derivatives	Amino acids, peptides, and analogues
pos.M267T556	0.169859221	MetaG7	Tributyl phosphate	Organic acids and derivatives	Carboxylic acids and derivatives	NA
neg.M480T648	0.16977566	MetaG3	LysoPE(18:0/0:0)	Lipids and lipid-like molecules	Glycerophospholipids	NA
pos.M319T244	0.168811993	MetaG7	Exemestane	Lipids and lipid-like molecules	Sterol Lipids	Androstane steroids
neg.M121T265	0.168255167	MetaG7	2-Hydroxybenzaldehyde	Benzenoids	Benzene and substituted derivatives	Carbonyl compounds
pos.M276T240	0.167882813	MetaG6	Ala-Trp	Organic acids and derivatives	Carboxylic acids and derivatives	Amino acids, peptides, and analogues
pos.M307T706	0.167318956	MetaG3	11,14,17-Eicosatrienoic acid, (Z,Z,Z)-	Lipids and lipid-like molecules	Fatty Acyls	Fatty acids and conjugates
pos.M889T632	0.167153917	MetaG7	HemiBMP 42:3; HemiBMP(12:0/14:0/16:3)	Lipids and lipid-like molecules	Glycerophospholipids	NA
neg.M465T404	0.166992398	MetaG11	Androstan-3-ol-17-one 3-glucuronide	Lipids and lipid-like molecules	Sterol Lipids	Steroidal glycosides
neg.M312T158	0.16660384	MetaG6	Adenosine	ıcleosides, nucleotides, and analogu	Purine nucleosides	NA
neg.M616T581	0.16628803	MetaG3	LysoPC 22:4	Lipids and lipid-like molecules	Glycerophospholipids	NA
pos.M231T138	0.166097034	MetaG4	Ile-Val	Organic acids and derivatives	Carboxylic acids and derivatives	Amino acids, peptides, and analogues
pos.M344T479_1	0.164664879	MetaG4	Dodecanoylcarnitine	Lipids and lipid-like molecules	Fatty Acyls	Fatty acid esters
pos.M269T119	0.163456977	MetaG6	His-Ile	Organic acids and derivatives	Carboxylic acids and derivatives	Amino acids, peptides, and analogues
neg.M522T597	0.162407818	MetaG11	LysoPS 18:1; LysoPS 18:1	Lipids and lipid-like molecules	Glycerophospholipids	NA

neg.M346T87	0.1622896	MetaG4	Adenosine 3'-monophosphate	ucleosides, nucleotides, and analogu	Ribonucleoside 3'Unknownphosphates	NA
pos.M233T153_1	0.160989404	MetaG4	Val-Asp	Organic acids and derivatives	Carboxylic acids and derivatives	Amino acids, peptides, and analogues
neg.M522T577	0.16052952	MetaG11	LysoPS 18:1; LysoPS 18:1	Lipids and lipid-like molecules	Glycerophospholipids	NA
pos.M281T178	0.159715295	MetaG4	Phe-Asp	Organic acids and derivatives	Carboxylic acids and derivatives	Amino acids, peptides, and analogues
pos.M300T661	0.15870661	MetaG7	Palmitoyl ethanolamide	Organic acids and derivatives	Carboximidic acids and derivatives	Carboximidic acids
pos.M260T290	0.157188536	MetaG4	Hexanoyl-L-carnitine	Lipids and lipid-like molecules	Fatty Acyls	Fatty acid esters
neg.M401T113	0.156390914	MetaG7	-O-.beta.-Galactopyranosyl-D-mannopyranos	Unknown	Unknown	NA
pos.M213T124	0.156050072	MetaG4	Pro-Pro	Unknown	Unknown	NA
neg.M271T377	0.155825475	MetaG4	Naringenin chalcone	Phenylpropanoids and polyketides	Linear 1,3Unknowndiarylpropanoids	Chalcones and dihydrochalcones
neg.M194T499	0.153964088	MetaG1	O-Benzyl-L-serine	Unknown	Unknown	NA
neg.M328T165_1	0.153349719	MetaG1	Guanosine	ucleosides, nucleotides, and analogu	Purine nucleosides	NA
pos.M182T170	0.15307486	MetaG4	L-Tyrosine	Organic acids and derivatives	Carboxylic acids and derivatives	Amino acids, peptides, and analogues
neg.M193T435	0.153025793	MetaG1	2,6-Dimethoxy-4-(1-propenyl)phenol	Benzenoids	Benzene and substituted derivatives	Methoxyphenols
neg.M520T530	0.151993544	MetaG11	LysoPS 18:2; LysoPS 18:2	Lipids and lipid-like molecules	Glycerophospholipids	NA
pos.M310T129	0.151722988	MetaG6	Tyr-Gln	Organic acids and derivatives	Carboxylic acids and derivatives	Amino acids, peptides, and analogues
pos.M279T597	0.149395824	MetaG5	Dibutyl phthalate	Benzenoids	Benzene and substituted derivatives	Benzoic acids and derivatives
pos.M508T603	0.149373756	MetaG3	(1Z-Octadecenyl)-sn-glycero-3-phosphocholi	Lipids and lipid-like molecules	Glycerophospholipids	NA
pos.M133T43	0.148273607	MetaG4	DL-Ornithine	Unknown	Unknown	Unknown
neg.M483T630	0.14799299	MetaG2	LysoPG 16:0; LysoPG 16:0	Lipids and lipid-like molecules	Glycerophospholipids	NA
neg.M564T533	0.147900905	MetaG8	LysoPC 18:2	Lipids and lipid-like molecules	Glycerophospholipids	Glycerophosphocholines
pos.M280T211	0.147568184	MetaG6	Phe-Gly-Gly	Unknown	Unknown	NA
pos.M299T467	0.147540655	MetaG7	19-Norepiandrosterone	Lipids and lipid-like molecules	Sterol Lipids	NA
pos.M126T47	0.145001044	MetaG2	N-Formylglycine	Unknown	Unknown	NA
neg.M147T287	0.144250218	MetaG4	p-Coumaraldehyde	Phenylpropanoids and polyketides	Cinnamic acids and derivatives	NA
pos.M684T72	0.143695825	MetaG1	Maltotetraose	Organic oxygen compounds	Organooxygen compounds	arbohydrates and carbohydrate conjugate
pos.M195T231	0.143684811	MetaG7	Caffeine	Organoheterocyclic compounds	Imidazopyrimidines	Purines and purine derivatives
pos.M283T620	0.143670782	MetaG6	trans-Vaccenic acid	Lipids and lipid-like molecules	Fatty Acyls	Fatty acids and conjugates
pos.M1071T769	0.143526422	MetaG10	HemiBMP 56:10; HemiBMP(16:2/20:4/20:4)	Lipids and lipid-like molecules	Glycerophospholipids	NA
pos.M343T438	0.142916755	MetaG4	CocamidopropylBetaine	Unknown	Unknown	NA
pos.M137T158	0.142889192	MetaG1	Hypoxanthine	Organoheterocyclic compounds	Imidazopyrimidines	Purines and purine derivatives

pos.M255T555	0.142564264	MetaG6	cis-9-Hexadecenoic acid	Lipids and lipid-like molecules	Fatty Acyls	Fatty acids and conjugates
pos.M803T736	0.141030947	MetaG10	MGDG 36:1; MGDG(18:0/18:1)	Lipids and lipid-like molecules	Glycerolipids	NA
neg.M546T553	0.140998811	MetaG11	LysoPS 20:3; LysoPS 20:3	Lipids and lipid-like molecules	Glycerophospholipids	NA
pos.M277T574	0.14060253	MetaG2	Kinoprene	Unknown	Unknown	NA
pos.M203T406	0.139654687	MetaG5	Diethyl adipate	Unknown	Unknown	NA
pos.M269T228	0.139530962	MetaG4	Cysteinyl-Phenylalanine	Organic acids and derivatives	Carboxylic acids and derivatives	Amino acids, peptides, and analogues
pos.M573T161	0.139168607	MetaG9	Adenylyl(3'-5')cytidine	ıcleosides, nucleotides, and analogu	Purine nucleotides	NA
neg.M147T183	0.138589945	MetaG4	Di-2-furanylmethane	Organoheterocyclic compounds	Heteroaromatic compounds	NA
pos.M703T645	0.137192638	MetaG10	Plasmenyl-PC 31:1; PC(P-14:0/17:1)	Lipids and lipid-like molecules	Glycerophospholipids	NA
pos.M152T162	0.137087079	MetaG1	Vigabatrin	Organic acids and derivatives	Carboxylic acids and derivatives	Amino acids, peptides, and analogues
pos.M137T95	0.13697983	MetaG1	Hypoxanthine	Organoheterocyclic compounds	Imidazopyrimidines	Purines and purine derivatives
pos.M223T214_2	0.135041301	MetaG6	Phe-Gly	Organic acids and derivatives	Carboxylic acids and derivatives	Amino acids, peptides, and analogues
pos.M427T680	0.134981497	MetaG7	Leupeptin	Organic acids and derivatives	Carboxylic acids and derivatives	NA
pos.M510T609_1	0.134744595	MetaG7	.-Heptadecanoyl-sn-glycero-3-phosphocholin	Lipids and lipid-like molecules	Glycerophospholipids	NA
pos.M275T49	0.134585164	MetaG6	Gln-Gln	Organic acids and derivatives	Carboxylic acids and derivatives	Amino acids, peptides, and analogues
pos.M667T52	0.134036608	MetaG1	Maltotetraose	Organic oxygen compounds	Organooxygen compounds	arbohydrates and carbohydrate conjugate
neg.M590T556	0.133946408	MetaG8	LysoPC 20:3	Lipids and lipid-like molecules	Glycerophospholipids	NA
pos.M162T49	0.133736014	MetaG4	L-Carnitine	Organic nitrogen compounds	Organonitrogen compounds	Quaternary ammonium salts
pos.M245T244	0.133212228	MetaG4	Isoleucyl-Leucine	Organic acids and derivatives	Carboxylic acids and derivatives	Amino acids, peptides, and analogues
neg.M313T543	0.132586741	MetaG7	Prexanthoperol	Lipids and lipid-like molecules	Prenol lipids	Diterpenoids
pos.M454T571	0.132492672	MetaG3	ıitoyl-2-hydroxy-sn-glycero-3-phosphoethanc	Lipids and lipid-like molecules	Glycerophospholipids	Glycerophosphoethanolamines
pos.M338T802	0.132293193	MetaG2	Erucamide	Unknown	Unknown	NA
pos.M398T544	0.132107756	MetaG4	Acylcarnitine 16:1	Lipids and lipid-like molecules	Fatty Acyls	Fatty acid esters
pos.M496T573	0.131983242	MetaG3	LysoPC 16:0	Lipids and lipid-like molecules	Glycerophospholipids	NA
pos.M283T172	0.131844824	MetaG4	Thr-Tyr	Organic acids and derivatives	Carboxylic acids and derivatives	Amino acids, peptides, and analogues
pos.M104T46	0.13169927	MetaG1	Choline	Organic nitrogen compounds	Organonitrogen compounds	Quaternary ammonium salts
neg.M464T541	0.131694563	MetaG8	LysoPE 17:1	Lipids and lipid-like molecules	Glycerophospholipids	NA
pos.M233T134	0.131615149	MetaG6	Val-Asp	Organic acids and derivatives	Carboxylic acids and derivatives	Amino acids, peptides, and analogues
neg.M259T49_2	0.131239407	MetaG2	2-Deoxyribose 5-phosphate	Organic oxygen compounds	Organooxygen compounds	arbohydrates and carbohydrate conjugate
neg.M227T75	0.131116936	MetaG4	Phosphoenolpyruvic acid	Organic acids and derivatives	rganic phosphoric acids and derivative	Phosphate esters

pos.M428T623	0.129546789	MetaG4	Stearoyl-L-carnitine	Lipids and lipid-like molecules	Fatty Acyls	Fatty acid esters
pos.M281T220_1	0.128994136	MetaG6	Phe-Asp	Organic acids and derivatives	Carboxylic acids and derivatives	Amino acids, peptides, and analogues
pos.M146T315	0.128959341	MetaG5	3-Formylindole	Organoheterocyclic compounds	Indoles and derivatives	Indoles
pos.M234T47	0.128606605	MetaG6	Gln-Ser	Organic acids and derivatives	Carboxylic acids and derivatives	Amino acids, peptides, and analogues
pos.M166T182_2	0.128442369	MetaG4	Phenylalanine	Organic acids and derivatives	Carboxylic acids and derivatives	Amino acids, peptides, and analogues
neg.M512T495	0.12789554	MetaG8	LysoPC 14:0	Lipids and lipid-like molecules	Glycerophospholipids	Glycerophosphocholines
pos.M175T45	0.127782488	MetaG6	Arginine	Organic acids and derivatives	Carboxylic acids and derivatives	Amino acids, peptides, and analogues
pos.M295T216	0.127655438	MetaG4	Isoleucyl-Tyrosine	Organic acids and derivatives	Carboxylic acids and derivatives	Amino acids, peptides, and analogues
neg.M565T60	0.127507275	MetaG9	UDP-D-glucose	ucleosides, nucleotides, and analogu	NA	Pyrimidine nucleotide sugars
neg.M207T280	0.127321993	MetaG10	2-Naphthalenesulfonic acid	Organosulfur compounds	Unknown	NA
neg.M413T351	0.126953892	MetaG3	alpha.-Pregnan-3.alpha.,17-diol-20-one 3-sulfa	Unknown	Unknown	NA
pos.M302T267	0.126898445	MetaG2	Ile-Gly-Ile	Organic acids and derivatives	Carboxylic acids and derivatives	NA
pos.M136T78_1	0.126881676	MetaG4	Adenine	Organoheterocyclic compounds	Imidazopyrimidines	Purines and purine derivatives
neg.M664T160	0.12676218	MetaG9	NADH	ucleosides, nucleotides, and analogu	NA	NA
pos.M915T746	0.126740007	MetaG9	HemiBMP 44:4; HemiBMP(12:0/16:2/16:2)	Lipids and lipid-like molecules	Glycerophospholipids	NA
pos.M223T196	0.126174492	MetaG6	Phe-Gly	Organic acids and derivatives	Carboxylic acids and derivatives	Amino acids, peptides, and analogues
neg.M221T566	0.126117256	MetaG1	taH,11xi)-11-Hydroxy-13-nor-6-eremophilen-	Lipids and lipid-like molecules	Prenol lipids	Sesquiterpenoids
pos.M231T40_2	0.12599089	MetaG5	Pro-Asp	Organic acids and derivatives	Carboxylic acids and derivatives	Amino acids, peptides, and analogues
pos.M321T634	0.125551176	MetaG4	8(9)-Epoxy-5Z,11Z,14Z-eicosatrienoic acid	Lipids and lipid-like molecules	Fatty Acyls	NA
pos.M180T231	0.125304876	MetaG4	3,4-Methylenedioxyamphetamine	Organoheterocyclic compounds	Benzodioxoles	NA
pos.M426T587	0.125088174	MetaG4	Acylcarnitine 18:1	Lipids and lipid-like molecules	Fatty Acyls	Fatty acid esters
neg.M183T280	0.125060759	MetaG1	2-Hydroxybenzyl alcohol	Benzenoids	Benzene and substituted derivatives	Benzyl alcohols
pos.M733T615	0.124976174	MetaG10	PC(16:0/16:1(9Z))	Lipids and lipid-like molecules	Glycerophospholipids	Glycerophosphocholines
neg.M494T506	0.124975059	MetaG11	LysoPS 16:1; LysoPS 16:1	Lipids and lipid-like molecules	Glycerophospholipids	NA
pos.M372T534	0.12415845	MetaG4	Acylcarnitine 14:0	Lipids and lipid-like molecules	Fatty Acyls	Fatty acid esters
pos.M775T692	0.123589268	MetaG10	PC(20:1(11Z)/15:0)	Lipids and lipid-like molecules	Glycerophospholipids	Glycerophosphocholines
pos.M338T99	0.123578901	MetaG6	Tyr-Arg	Organic acids and derivatives	Carboxylic acids and derivatives	Amino acids, peptides, and analogues
neg.M548T595	0.123314157	MetaG11	LysoPS 20:2; LysoPS 20:2	Lipids and lipid-like molecules	Glycerophospholipids	NA
neg.M568T532	0.122835441	MetaG11	LysoPS 22:6; LysoPS 22:6	Lipids and lipid-like molecules	Glycerophospholipids	NA
neg.M201T77	0.122810982	MetaG4	cis,cis-Muconic acid	Lipids and lipid-like molecules	Fatty Acyls	Fatty acids and conjugates

pos.M282T177	0.122622046	MetaG6	3'-O-Methyladenosine	ucleosides, nucleotides, and analogu	Purine nucleosides	NA
pos.M175T135	0.122551852	MetaG6	Val-Gly	Organic acids and derivatives	Carboxylic acids and derivatives	Amino acids, peptides, and analogues
pos.M747T642	0.121368321	MetaG10	PC(15:0/18:1(11Z))	Lipids and lipid-like molecules	Glycerophospholipids	Glycerophosphocholines
pos.M251T503	0.121367802	MetaG5	3,5-Di-tert-butyl-4-hydroxybenzoic acid	Benzenoids	Benzene and substituted derivatives	NA
neg.M227T53	0.12092704	MetaG4	Phospho(enol)pyruvic acid	Organic acids and derivatives	rganic phosphoric acids and derivative	Phosphate esters
pos.M269T157	0.120501869	MetaG1	Inosine	ucleosides, nucleotides, and analogu	Purine nucleosides	NA
neg.M512T507	0.120316442	MetaG3	LysoPC 14:0	Lipids and lipid-like molecules	Glycerophospholipids	Glycerophosphocholines
pos.M130T48	0.119697167	MetaG4	L-5-Oxoproline	Organic acids and derivatives	Carboxylic acids and derivatives	Amino acids, peptides, and analogues
pos.M118T75	0.119689372	MetaG4	2-Ethylacrylic acid	Lipids and lipid-like molecules	Fatty Acyls	Fatty acids and conjugates
pos.M148T48	0.119487773	MetaG4	Glutamic acid	Organic acids and derivatives	Carboxylic acids and derivatives	Amino acids, peptides, and analogues
neg.M466T578	0.119442541	MetaG3	LysoPE 17:0	Lipids and lipid-like molecules	Glycerophospholipids	NA
pos.M189T96	0.118915463	MetaG4	Ala-Val	Unknown	null	Amino acids, peptides, and analogues
neg.M437T445	0.118369593	MetaG9	Ursodeoxycholic acid	Lipids and lipid-like molecules	Sterol Lipids	Bile acids, alcohols and derivatives
pos.M249T189	0.118128984	MetaG4	Valyl-Methionine	Organic acids and derivatives	Carboxylic acids and derivatives	Amino acids, peptides, and analogues
neg.M452T571	0.117117076	MetaG3	LysoPE(16:0/0:0)	Lipids and lipid-like molecules	Glycerophospholipids	Glycerophosphoethanolamines
pos.M118T54	0.117055982	MetaG4	Betaine	Organic acids and derivatives	Carboxylic acids and derivatives	Amino acids, peptides, and analogues
pos.M263T214	0.116693326	MetaG4	Isoleucyl-Methionine	Organic acids and derivatives	Carboxylic acids and derivatives	Amino acids, peptides, and analogues
pos.M253T52_2	0.116130642	MetaG4	His-Pro	Organic acids and derivatives	Carboxylic acids and derivatives	Amino acids, peptides, and analogues
pos.M136T52_1	0.115693323	MetaG4	Adenine	Organoheterocyclic compounds	Imidazopyrimidines	Purines and purine derivatives
pos.M302T259	0.114960917	MetaG4	Trp-Pro	Organic acids and derivatives	Carboxylic acids and derivatives	Amino acids, peptides, and analogues
pos.M288T359	0.114850523	MetaG7	Octanoylcarnitine	Lipids and lipid-like molecules	Fatty Acyls	Fatty acid esters
neg.M542T528	0.113563478	MetaG11	LysoPS 20:5; LysoPS 20:5	Lipids and lipid-like molecules	Glycerophospholipids	NA
pos.M310T171	0.113431016	MetaG6	Gln-Tyr	Organic acids and derivatives	Carboxylic acids and derivatives	Amino acids, peptides, and analogues
pos.M181T206	0.11318424	MetaG7	Aminophylline	Organic nitrogen compounds	Organonitrogen compounds	Unknown
neg.M178T219	0.112773698	MetaG7	Cyclamate	Organic acids and derivatives	Sulfamic acid derivatives	Cyclamates
pos.M112T41	0.112301447	MetaG2	Histamine	Organic nitrogen compounds	Organonitrogen compounds	Amines
neg.M508T539	0.112070901	MetaG11	LysoPS 17:1; LysoPS 17:1	Lipids and lipid-like molecules	Glycerophospholipids	NA
pos.M246T104	0.111324644	MetaG4	Isoleucyl-Asparagine	Organic acids and derivatives	Carboxylic acids and derivatives	Amino acids, peptides, and analogues
neg.M198T213	0.11127085	MetaG1	D-p-Chlorophenylalanine	Organic acids and derivatives	Carboxylic acids and derivatives	NA
pos.M136T52_2	0.111026986	MetaG6	Adenine	Organoheterocyclic compounds	Imidazopyrimidines	Purines and purine derivatives

neg.M507T577	0.110366512	MetaG10	LysoPG 18:2; LysoPG 18:2	Lipids and lipid-like molecules	Glycerophospholipids	NA
pos.M276T46	0.110084973	MetaG6	Lys-Glu	Organic acids and derivatives	Carboxylic acids and derivatives	Amino acids, peptides, and analogues
pos.M510T609_2	0.109997598	MetaG7	.-Heptadecanoyl-sn-glycero-3-phosphocholin	Lipids and lipid-like molecules	Glycerophospholipids	NA
pos.M705T555	0.109951908	MetaG10	PC(14:1(9Z)/16:0)	Lipids and lipid-like molecules	Glycerophospholipids	Glycerophosphocholines
pos.M231T96	0.109821085	MetaG4	Asp-Pro	Organic acids and derivatives	Carboxylic acids and derivatives	Amino acids, peptides, and analogues
pos.M170T525	0.109396742	MetaG5	Diphenylamine	Benzenoids	Benzene and substituted derivatives	Aniline and substituted anilines
neg.M568T649	0.109233598	MetaG3	LysoPC 18:0	Lipids and lipid-like molecules	Glycerophospholipids	Glycerophosphocholines
neg.M588T534	0.108917445	MetaG6	LysoPC 20:4	Lipids and lipid-like molecules	Glycerophospholipids	Glycerophosphocholines
neg.M503T123	0.108616763	MetaG6	1-Kestose	Organic oxygen compounds	Organooxygen compounds	arbohydrates and carbohydrate conjugate
pos.M524T645	0.107965931	MetaG3	itearoyl-2-hydroxy-sn-glycero-3-phosphocho	Lipids and lipid-like molecules	Glycerophospholipids	Glycerophosphocholines
neg.M592T602	0.10793055	MetaG3	LysoPC 20:2	Lipids and lipid-like molecules	Glycerophospholipids	NA
pos.M256T684	0.107829231	MetaG7	Palmitamide	Lipids and lipid-like molecules	Fatty Acyls	Fatty amides
pos.M310T744	0.107799351	MetaG2	Oleoyl ethylamide	Unknown	Unknown	NA
pos.M522T588	0.107320572	MetaG3	1-Oleoyl-sn-glycero-3-phosphocholine	Lipids and lipid-like molecules	Glycerophospholipids	Glycerophosphocholines
pos.M294T175	0.107093747	MetaG4	Phenylalanyl-Gamma-glutamate	Organic acids and derivatives	Carboxylic acids and derivatives	Amino acids, peptides, and analogues
pos.M835T637	0.10699417	MetaG10	PC(22:5(4Z,7Z,10Z,13Z,16Z)/18:1(9Z))	Lipids and lipid-like molecules	Glycerophospholipids	Glycerophosphocholines
pos.M357T699	0.106164568	MetaG8	Monoelaidin	Lipids and lipid-like molecules	Glycerolipids	Monoradylglycerols
pos.M173T53	0.105437192	MetaG4	Gly-Pro	Organic acids and derivatives	Carboxylic acids and derivatives	Amino acids, peptides, and analogues
pos.M926T763	0.105241747	MetaG9	TG 57:15; TG(19:5/19:5/19:5)	Lipids and lipid-like molecules	Glycerolipids	NA
pos.M258T48	0.105199085	MetaG4	Glycerophosphocholine	Lipids and lipid-like molecules	Glycerophospholipids	Glycerophosphocholines
neg.M606T53	0.105050954	MetaG9	Uridine 5'-diphospho-N-acetylgalactosamine	ıcleosides, nucleotides, and analogu	Pyrimidine nucleotides	Pyrimidine nucleotide sugars
pos.M1043T745	0.104977916	MetaG10	HemiBMP 54:10; HemiBMP(16:2/16:2/22:6)	Lipids and lipid-like molecules	Glycerophospholipids	NA
neg.M215T119	0.104963586	MetaG4	1,4,5-Trimethyl-naphtalene	Benzenoids	Naphthalenes	NA
neg.M244T48_2	0.104285643	MetaG4	Iminoerythrose 4-phosphate	Organic acids and derivatives	rganic phosphoric acids and derivative	Phosphate esters
pos.M308T92	0.103874955	MetaG4	L-Glutathione, reduced	Organic acids and derivatives	Carboxylic acids and derivatives	Amino acids, peptides, and analogues
pos.M195T486	0.103602433	MetaG5	Sedanolide	Organoheterocyclic compounds	Lactones	NA
pos.M223T463	0.103517182	MetaG5	Diethyl phthalic acid	Benzenoids	Benzene and substituted derivatives	Benzoic acids and derivatives
pos.M295T221	0.103369048	MetaG6	Aspartame	Organic acids and derivatives	Carboxylic acids and derivatives	Amino acids, peptides, and analogues
pos.M379T649	0.103299679	MetaG4	1-Arachidonoylglycerol	Lipids and lipid-like molecules	Glycerolipids	Monoradylglycerols
pos.M130T43	0.102809231	MetaG4	L-Pipecolic acid	Organic acids and derivatives	Carboxylic acids and derivatives	Amino acids, peptides, and analogues

pos.M215T152	0.102245139	MetaG4	Val-Pro	Organic acids and derivatives	Carboxylic acids and derivatives	Amino acids, peptides, and analogues
neg.M552T543	0.101980504	MetaG8	LysoPC 17:1	Lipids and lipid-like molecules	Glycerophospholipids	NA
neg.M554T610	0.101884588	MetaG3	LysoPC 17:0	Lipids and lipid-like molecules	Glycerophospholipids	Glycerophosphocholines
pos.M120T47	0.101780195	MetaG4	L-Allothreonine	Organic acids and derivatives	Carboxylic acids and derivatives	Amino acids, peptides, and analogues
neg.M526T527	0.101704749	MetaG8	LysoPC 15:0	Lipids and lipid-like molecules	Glycerophospholipids	Glycerophosphocholines
pos.M217T173	0.100653549	MetaG4	Valyl-Valine	Organic acids and derivatives	Carboxylic acids and derivatives	Amino acids, peptides, and analogues
pos.M276T219	0.100633522	MetaG4	Trp-Ala	Organic acids and derivatives	Carboxylic acids and derivatives	Amino acids, peptides, and analogues
pos.M203T37	0.100476247	MetaG4	Spermine	Organic nitrogen compounds	Organonitrogen compounds	Amines
pos.M189T169	0.100271869	MetaG6	Linsidomine cation	Unknown	Unknown	NA
pos.M232T205	0.100074145	MetaG4	(R)-Butyrylcarnitine	Lipids and lipid-like molecules	Fatty Acyls	Fatty acid esters
pos.M229T208	0.099897775	MetaG4	Ile-Pro	Organic acids and derivatives	Carboxylic acids and derivatives	Amino acids, peptides, and analogues
neg.M124T48	0.099606512	MetaG2	Taurine	Organic acids and derivatives	Organic sulfonic acids and derivatives	Organosulfonic acids and derivatives
pos.M200T215	0.099554142	MetaG1	2-Chloro-L-phenylalanine	Organic acids and derivatives	Carboxylic acids and derivatives	NA
neg.M231T196	0.099041045	MetaG6	1,2-Dihydro-1,1,6-trimethylnaphthalene	Benzenoids	Naphthalenes	NA
neg.M526T544	0.098548825	MetaG6	LysoPE 22:5	Lipids and lipid-like molecules	Glycerophospholipids	Glycerophosphoethanolamines
pos.M205T116	0.09789981	MetaG6	Ser-Val	Organic acids and derivatives	Carboxylic acids and derivatives	Amino acids, peptides, and analogue
pos.M386T553	0.097689753	MetaG4	Acylcarnitine 15:0	Lipids and lipid-like molecules	Fatty Acyls	NA
pos.M269T166	0.097263113	MetaG6	Tyr-Ser	Organic acids and derivatives	Carboxylic acids and derivatives	Amino acids, peptides, and analogues
neg.M181T214	0.097139797	MetaG4	alpha-Methyl-2-furanacrolein	Organoheterocyclic compounds	Heteroaromatic compounds	NA
pos.M156T44	0.096805381	MetaG4	L-Histidine	Organic acids and derivatives	Carboxylic acids and derivatives	Amino acids, peptides, and analogues
pos.M251T157	0.096549634	MetaG4	Thr-Met	Organic acids and derivatives	Carboxylic acids and derivatives	Amino acids, peptides, and analogues
pos.M235T207	0.096536084	MetaG4	Cys-Ile	Organic acids and derivatives	Carboxylic acids and derivatives	Amino acids, peptides, and analogues
pos.M203T52_1	0.096410426	MetaG4	Ser-Pro	Organic acids and derivatives	Carboxylic acids and derivatives	Amino acids, peptides, and analogues
pos.M316T419	0.096403654	MetaG7	Decanoyl-L-carnitine	Lipids and lipid-like molecules	Fatty Acyls	Fatty acid esters
pos.M280T164	0.096383801	MetaG4	Phe-Asn	Organic acids and derivatives	Carboxylic acids and derivatives	Amino acids, peptides, and analogues
pos.M789T768	0.095679568	MetaG9	PC(22:1(13Z)/14:0)	Lipids and lipid-like molecules	Glycerophospholipids	Glycerophosphocholines
pos.M246T193	0.094674401	MetaG6	Isoleucyl-Asparagine	Organic acids and derivatives	Carboxylic acids and derivatives	Amino acids, peptides, and analogues
pos.M246T132	0.094426253	MetaG6	Valyl-Gamma-glutamate	Organic acids and derivatives	Carboxylic acids and derivatives	Amino acids, peptides, and analogues
pos.M195T515	0.094352835	MetaG5	Butyl 4-hydroxybenzoate	Benzenoids	Benzene and substituted derivatives	Benzoic acids and derivatives
neg.M502T555	0.094237112	MetaG6	LysoPE 20:3	Lipids and lipid-like molecules	Glycerophospholipids	Glycerophosphoethanolamines

neg.M936T366	0.093781368	MetaG7	HemiBMP 48:12; HemiBMP(16:4/16:4/16:4)	Lipids and lipid-like molecules	Glycerophospholipids	NA
pos.M304T256	0.093587987	MetaG4	Val-Trp	Organic acids and derivatives	Carboxylic acids and derivatives	Amino acids, peptides, and analogues
pos.M329T252	0.093187695	MetaG4	Tyrosyl-Phenylalanine	Organic acids and derivatives	Carboxylic acids and derivatives	Amino acids, peptides, and analogues
neg.M448T443	0.093176868	MetaG3	Glycoursodeoxycholic acid	Lipids and lipid-like molecules	Sterol Lipids	Bile acids, alcohols and derivatives
pos.M215T188	0.092190104	MetaG4	Val-Pro	Organic acids and derivatives	Carboxylic acids and derivatives	Amino acids, peptides, and analogues
pos.M206T300	0.09210676	MetaG4	DL-Indole-3-lactic acid	Organoheterocyclic compounds	Indoles and derivatives	Indolyl carboxylic acids and derivatives
pos.M281T579	0.091507743	MetaG4	Linoleic acid	Lipids and lipid-like molecules	Fatty Acyls	Lineolic acids and derivatives
pos.M270T111	0.091296149	MetaG6	His-Asn	Organic acids and derivatives	Carboxylic acids and derivatives	Amino acids, peptides, and analogues
neg.M538T513	0.090964277	MetaG8	LysoPC 16:1	Lipids and lipid-like molecules	Glycerophospholipids	Glycerophosphocholines
pos.M233T53	0.090312847	MetaG4	Val-Asp	Organic acids and derivatives	Carboxylic acids and derivatives	Amino acids, peptides, and analogues
neg.M159T373	0.090139747	MetaG7	1-Methylcyclohexanol	Organic oxygen compounds	Organooxygen compounds	Alcohols and polyols
pos.M252T154	0.090098129	MetaG6	Cordycepin	Unknown	Unknown	NA
pos.M147T43	0.089960787	MetaG4	L-Pipecolic acid	Organic acids and derivatives	Carboxylic acids and derivatives	Amino acids, peptides, and analogues
neg.M233T560	0.089632034	MetaG10	3,5-Di-tert-butyl-2-hydroxybenzaldehyde	Unknown	Unknown	NA
pos.M436T770	0.089366447	MetaG5	Di(2-nonyl) phthalate	Unknown	Unknown	NA
pos.M215T173	0.088849877	MetaG4	Val-Pro	Organic acids and derivatives	Carboxylic acids and derivatives	Amino acids, peptides, and analogues
pos.M239T160	0.08879335	MetaG6	Tyr-Gly	Organic acids and derivatives	Carboxylic acids and derivatives	NA
pos.M189T123	0.088719582	MetaG6	Ala-Val	Unknown	null	Amino acids, peptides, and analogues
pos.M116T51	0.088711112	MetaG4	1-Amino-1-cyclobutanecarboxylic acid	Unknown	Unknown	NA
pos.M448T555	0.088616432	MetaG9	Acylcarnitine 20:4	Lipids and lipid-like molecules	Fatty Acyls	NA
pos.M282T692	0.088335763	MetaG7	Oleamide	Lipids and lipid-like molecules	Fatty Acyls	Fatty amides
pos.M355T657	0.087958876	MetaG8	1-Monolinoleoyl-rac-glycerol	Lipids and lipid-like molecules	Fatty Acyls	Lineolic acids and derivatives
pos.M237T194	0.087175411	MetaG6	Phenylalanyl-Alanine	Organic acids and derivatives	Carboxylic acids and derivatives	Amino acids, peptides, and analogues
neg.M526T563	0.087135061	MetaG6	LysoPE 22:5	Lipids and lipid-like molecules	Glycerophospholipids	Glycerophosphoethanolamines
pos.M182T127	0.086811477	MetaG4	L-Tyrosine	Organic acids and derivatives	Carboxylic acids and derivatives	Amino acids, peptides, and analogues
pos.M295T236	0.086778765	MetaG6	Tyr-Ile	Organic acids and derivatives	Carboxylic acids and derivatives	Amino acids, peptides, and analogues
pos.M152T52	0.086069541	MetaG1	Guanine	Organoheterocyclic compounds	Imidazopyrimidines	Purines and purine derivatives
pos.M295T187	0.085406306	MetaG4	Aspartame	Organic acids and derivatives	Carboxylic acids and derivatives	Amino acids, peptides, and analogues
neg.M554T581	0.085232787	MetaG8	LysoPC 17:0	Lipids and lipid-like molecules	Glycerophospholipids	Glycerophosphocholines
pos.M889T579	0.084765033	MetaG7	HemiBMP 42:3; HemiBMP(12:0/14:0/16:3)	Lipids and lipid-like molecules	Glycerophospholipids	NA

neg.M526T539	0.084437311	MetaG3	LysoPC 15:0	Lipids and lipid-like molecules	Glycerophospholipids	Glycerophosphocholines
neg.M342T204_1	0.084367177	MetaG9	5'-Methylthioadenosine	ucleosides, nucleotides, and analogu	5'Unknowndeoxyribonucleosides	5'-deoxy-5'-thionucleosides
neg.M349T120	0.08424567	MetaG6	D-Sedoheptulose 7-phosphate	Organic oxygen compounds	Organooxygen compounds	arbohydrates and carbohydrate conjugate
pos.M247T142	0.083900225	MetaG6	Isoleucyl-Aspartate	Organic acids and derivatives	Carboxylic acids and derivatives	Amino acids, peptides, and analogues
pos.M147T47	0.083794219	MetaG4	D-Pyroglutamic acid	Organic acids and derivatives	Carboxylic acids and derivatives	Amino acids, peptides, and analogues
neg.M307T51	0.083581137	MetaG4	Mevalonic acid 5-pyrophosphate	Organic oxygen compounds	Organic oxoanionic compounds	Organic pyrophosphates
pos.M237T217	0.083221968	MetaG6	Phe-Ala	Organic acids and derivatives	Carboxylic acids and derivatives	Amino acids, peptides, and analogues
pos.M306T239	0.082981598	MetaG4	Thr-Trp	Organic acids and derivatives	Carboxylic acids and derivatives	Amino acids, peptides, and analogues
neg.M209T394	0.082902281	MetaG9	n-Octyl sulfate	Unknown	Unknown	NA
pos.M233T78	0.082687963	MetaG4	Val-Asp	Organic acids and derivatives	Carboxylic acids and derivatives	Amino acids, peptides, and analogues
pos.M508T545	0.082138776	MetaG8	Plasmenyl-PC 17:0; PC(P-14:0/3:0)	Lipids and lipid-like molecules	Glycerophospholipids	NA
pos.M253T210	0.082059262	MetaG6	Ser-Phe	Organic acids and derivatives	Carboxylic acids and derivatives	Amino acids, peptides, and analogues
pos.M203T160_2	0.082054706	MetaG6	Isoleucyl-Alanine	Organic acids and derivatives	Carboxylic acids and derivatives	Amino acids, peptides, and analogues
pos.M217T116	0.081521488	MetaG4	Pro-Thr	Organic acids and derivatives	Carboxylic acids and derivatives	Amino acids, peptides, and analogues
pos.M175T64	0.08149683	MetaG4	N-.alpha.-Acetyl-L-ornithine	Organic acids and derivatives	Carboxylic acids and derivatives	Amino acids, peptides, and analogues
neg.M203T214	0.08119908	MetaG4	5-Methylquinoxaline	Organoheterocyclic compounds	Naphthyridines	Benzodiazines
pos.M267T217	0.081017533	MetaG4	Thr-Phe	Organic acids and derivatives	Carboxylic acids and derivatives	Amino acids, peptides, and analogues
neg.M606T67	0.080951434	MetaG9	Uridine 5'-diphospho-N-acetylgalactosamine	ucleosides, nucleotides, and analogu	Unknown	NA
pos.M326T169	0.080933719	MetaG6	Bisdemethoxycurcumin	Phenylpropanoids and polyketides	Diarylheptanoids	Linear diarylheptanoids
pos.M118T839_3	0.079577435	MetaG5	Betaine	Organic acids and derivatives	Carboxylic acids and derivatives	Amino acids, peptides, and analogues
pos.M368T257	0.07848106	MetaG4	Trp-Tyr	Organic acids and derivatives	Carboxylic acids and derivatives	Amino acids, peptides, and analogues
pos.M205T73	0.078061152	MetaG4	Val-Ser	Organic acids and derivatives	Carboxylic acids and derivatives	Amino acids, peptides, and analogues
pos.M279T193	0.077708101	MetaG4	Tyr-Pro	Organic acids and derivatives	Carboxylic acids and derivatives	Amino acids, peptides, and analogues
pos.M276T45	0.076539488	MetaG6	Lys-Glu	Organic acids and derivatives	Carboxylic acids and derivatives	Amino acids, peptides, and analogues
pos.M310T113	0.076226062	MetaG6	Tyr-Lys	Organic acids and derivatives	Carboxylic acids and derivatives	Amino acids, peptides, and analogues
pos.M303T168	0.075951597	MetaG6	His-Phe	Organic acids and derivatives	Carboxylic acids and derivatives	Amino acids, peptides, and analogues
neg.M528T579	0.075740124	MetaG6	LysoPE 22:4	Lipids and lipid-like molecules	Glycerophospholipids	Glycerophosphoethanolamines
pos.M331T182	0.075480976	MetaG4	Bestatin	Unknown	Unknown	NA
pos.M280T193	0.074086514	MetaG4	Phe-Asn	Organic acids and derivatives	Carboxylic acids and derivatives	Amino acids, peptides, and analogues
pos.M352T301	0.073667794	MetaG4	Tryptophyl-Phenylalanine	Organic acids and derivatives	Carboxylic acids and derivatives	Amino acids, peptides, and analogues

pos.M203T197	0.073608959	MetaG6	L-Alanyl-L-norleucine	Organic acids and derivatives	Carboxylic acids and derivatives	NA
pos.M838T766	0.073318679	MetaG9	TG 50:10; TG(12:3/18:4/20:3)	Lipids and lipid-like molecules	Glycerolipids	NA
pos.M510T580	0.072982678	MetaG8	.-Heptadecanoyl-sn-glycero-3-phosphocholin	Lipids and lipid-like molecules	Glycerophospholipids	NA
pos.M188T216	0.072856387	MetaG4	3-Indoleacrylic acid	Organoheterocyclic compounds	Indoles and derivatives	Indoles
pos.M134T47	0.072851365	MetaG4	D-Aspartic acid	Organic acids and derivatives	Carboxylic acids and derivatives	Amino acids, peptides, and analogues
pos.M522T576	0.072791934	MetaG8	Plasmenyl-PC 18:0; PC(P-14:0/4:0)	Lipids and lipid-like molecules	Glycerophospholipids	NA
pos.M265T238	0.072506325	MetaG4	Valyl-Phenylalanine	Organic acids and derivatives	Carboxylic acids and derivatives	Amino acids, peptides, and analogues
pos.M246T79	0.072436191	MetaG4	Valyl-Gamma-glutamate	Organic acids and derivatives	Carboxylic acids and derivatives	Amino acids, peptides, and analogues
pos.M520T538	0.071646387	MetaG8	LysoPC 18:2	Lipids and lipid-like molecules	Glycerophospholipids	Glycerophosphocholines
pos.M265T241	0.071083561	MetaG4	Valyl-Phenylalanine	Organic acids and derivatives	Carboxylic acids and derivatives	Amino acids, peptides, and analogues
pos.M281T190	0.070998005	MetaG4	Val-Tyr	Organic acids and derivatives	Carboxylic acids and derivatives	Amino acids, peptides, and analogues
pos.M207T41	0.069733373	MetaG4	Ser-Thr	Organic acids and derivatives	Carboxylic acids and derivatives	Amino acids, peptides, and analogues
pos.M253T170	0.069706849	MetaG6	Ala-Tyr	Organic acids and derivatives	Carboxylic acids and derivatives	Amino acids, peptides, and analogues
pos.M122T49	0.069703322	MetaG4	D-Cysteine	Organic acids and derivatives	Carboxylic acids and derivatives	Amino acids, peptides, and analogues
pos.M173T79_2	0.069672735	MetaG4	Gly-Pro	Organic acids and derivatives	Carboxylic acids and derivatives	Amino acids, peptides, and analogues
neg.M187T197	0.068566137	MetaG6	N6-Acetyl-L-lysine	Organic acids and derivatives	Carboxylic acids and derivatives	Amino acids, peptides, and analogues
pos.M263T230	0.068520986	MetaG4	Prolylphenylalanine	Organic acids and derivatives	Carboxylic acids and derivatives	Amino acids, peptides, and analogues
pos.M232T66	0.067839938	MetaG4	Valyl-Asparagine	Organic acids and derivatives	Carboxylic acids and derivatives	Amino acids, peptides, and analogues
pos.M233T197	0.067659453	MetaG6	Thr-Leu	Organic acids and derivatives	Carboxylic acids and derivatives	Amino acids, peptides, and analogues
pos.M234T42	0.067005504	MetaG6	Ser-Lys	Organic acids and derivatives	Carboxylic acids and derivatives	Amino acids, peptides, and analogues
pos.M263T249	0.066231919	MetaG4	Prolylphenylalanine	Organic acids and derivatives	Carboxylic acids and derivatives	Amino acids, peptides, and analogues
pos.M175T104	0.066120492	MetaG4	Val-Gly	Organic acids and derivatives	Carboxylic acids and derivatives	Amino acids, peptides, and analogues
pos.M203T79_2	0.065954075	MetaG4	Ser-Pro	Organic acids and derivatives	Carboxylic acids and derivatives	Amino acids, peptides, and analogues
pos.M260T137	0.065354807	MetaG6	Leu-Lys	Organic acids and derivatives	Carboxylic acids and derivatives	Amino acids, peptides, and analogues
pos.M205T216	0.064525238	MetaG4	3-Indoleacrylic acid	Organoheterocyclic compounds	Indoles and derivatives	Indoles
pos.M189T198	0.063948093	MetaG6	Leu-Gly	Organic acids and derivatives	Carboxylic acids and derivatives	Amino acids, peptides, and analogues
neg.M263T238	0.061580114	MetaG4	Suberoylanilide hydroxamic acid	Benzenoids	Benzene and substituted derivatives	NA
pos.M207T48	0.061265053	MetaG6	Ser-Thr	Organic acids and derivatives	Carboxylic acids and derivatives	Amino acids, peptides, and analogues
pos.M205T52	0.060345191	MetaG6	Val-Ser	Organic acids and derivatives	Carboxylic acids and derivatives	Amino acids, peptides, and analogues
pos.M231T221	0.057509576	MetaG4	Val-Leu	Organic acids and derivatives	Carboxylic acids and derivatives	Amino acids, peptides, and analogues

pos.M204T42	0.055675255	MetaG6	Gly-Lys	Organic acids and derivatives	Carboxylic acids and derivatives	Amino acids, peptides, and analogues
