# Supplementary material for: Metabolic profiles outperform the microbiota in assessing the response of vaginal microenvironments to the changed state of HPV infection
Source: NPJ Biofilms Microbiomes. 2024 Mar 20;10:26. doi: 10.1038/s41522-024-00500-0 (PMC10954630; doi:10.1038/s41522-024-00500-0)
Supplement: Supplementary file 2 — nr-reporting-summary [file 41522_2024_500_MOESM2_ESM.pdf]

nature portfolio

Corresponding author(s): Ruifang Wu
Last updated by author(s): Mar 1, 2024

Reporting Summary

Nature Portfolio wishes to improve the reproducibility of the work that we publish. This form provides structure for consistency and transparency in reporting. For further information on Nature Portfolio policies, see our Editorial Policies and the Editorial Policy Checklist.

Statistics

For all statistical analyses, confirm that the following items are present in the figure legend, table legend, main text, or Methods section.

n/a	Confirmed	
☐	☒	The exact sample size (*n*) for each experimental group/condition, given as a discrete number and unit of measurement
☐	☒	A statement on whether measurements were taken from distinct samples or whether the same sample was measured repeatedly
☐	☒	The statistical test(s) used AND whether they are one- or two-sided<br>*Only common tests should be described solely by name; describe more complex techniques in the Methods section.*
☐	☒	A description of all covariates tested
☐	☒	A description of any assumptions or corrections, such as tests of normality and adjustment for multiple comparisons
☐	☒	A full description of the statistical parameters including central tendency (e.g. means) or other basic estimates (e.g. regression coefficient) AND variation (e.g. standard deviation) or associated estimates of uncertainty (e.g. confidence intervals)
☐	☒	For null hypothesis testing, the test statistic (e.g. *F*, *t*, *r*) with confidence intervals, effect sizes, degrees of freedom and *P* value noted<br>*Give P values as exact values whenever suitable.*
☒	☐	For Bayesian analysis, information on the choice of priors and Markov chain Monte Carlo settings
☒	☐	For hierarchical and complex designs, identification of the appropriate level for tests and full reporting of outcomes
☒	☐	Estimates of effect sizes (e.g. Cohen's *d*, Pearson's *r*), indicating how they were calculated

Our web collection on statistics for biologists contains articles on many of the points above.

Software and code

Policy information about availability of computer code

Data collection	No software was used
Data analysis	R software (v.4.05)

For manuscripts utilizing custom algorithms or software that are central to the research but not yet described in published literature, software must be made available to editors and reviewers. We strongly encourage code deposition in a community repository (e.g. GitHub). See the Nature Portfolio guidelines for submitting code & software for further information.

Data

Policy information about availability of data

All manuscripts must include a data availability statement. This statement should provide the following information, where applicable:

- Accession codes, unique identifiers, or web links for publicly available datasets
- A description of any restrictions on data availability
- For clinical datasets or third party data, please ensure that the statement adheres to our policy

The data of 16S rDNA amplicon sequencing was submitted to CNGB Sequence Archive (CNSA) under Project No. CNP0004255 and CNP0002763. The annotated metabolomes datasets are available at the MetaboLights database (http://www.ebi.ac.uk/metabolights/) with accession number MTBLS7805. The authors declare that all other data supporting the findings of the study are available from the corresponding authors on reasonable request.

Research involving human participants, their data, or biological material

Policy information about studies with human participants or human data. See also policy information about sex, gender (identity/presentation), and sexual orientation and race, ethnicity and racism.

Reporting on sex and gender	Attenders of this study are females who received therapies for high-grade cervical intraepithelial noeplasia.
Reporting on race, ethnicity, or other socially relevant groupings	There were no race, ethnicity or other socially relevant groupings , as all attenders were Han ethnicity. We applied PERMANOVA to assess the confounding effects of various factors, like HPV genotypes, age, vaccination, on differences of vaginal microbiota and metabolome.
Population characteristics	See above.
Recruitment	Doctors recruited patients based on inclusion criteria, such as no genital infections and antibiotics exposure one month prior to the sampling.
Ethics oversight	Ethics Committee of Peking University Shenzhen Hospital.

Note that full information on the approval of the study protocol must also be provided in the manuscript.

Field-specific reporting

Please select the one below that is the best fit for your research. If you are not sure, read the appropriate sections before making your selection.

☒ Life sciences ☐ Behavioural & social sciences ☐ Ecological, evolutionary & environmental sciences

For a reference copy of the document with all sections, see nature.com/documents/nr-reporting-summary-flat.pdf

Life sciences study design

All studies must disclose on these points even when the disclosure is negative.

Sample size	The sample size is determined based on data available for high-grade CIN patients. Power calculation was performed as a justification for dimensionality reduction of the multi-omic features based on the sample size, to ensure adequate statistical power for integration analysis.
Data exclusions	No collected data were excluded.
Replication	There was no replication assessment, as this is a prospective study that investigated dynamic changes of vaginal mcirobiota and metabolome for high-grade CIN patients. However, findings in our study are concordant with previous reports on sensitive of functional blueprints to environmental changes.
Randomization	First, we allocated patients into three groups following therapy strategy. After PERMANOVA, included attenders were allocated to baseline and post-treatment group to analyzed dynamic changes post-therapy.
Blinding	Data analysts were blinded to group allocation during sample collection, processing and sequencing. Downstream multiomic analyses require the clinical data so blinding is not applicable.

Reporting for specific materials, systems and methods

We require information from authors about some types of materials, experimental systems and methods used in many studies. Here, indicate whether each material, system or method listed is relevant to your study. If you are not sure if a list item applies to your research, read the appropriate section before selecting a response.

Materials & experimental systems

n/a	Involved in the study	
☒	☐	Antibodies
☒	☐	Eukaryotic cell lines
☒	☐	Palaeontology and archaeology
☒	☐	Animals and other organisms
☐	☒	Clinical data
☒	☐	Dual use research of concern
☒	☐	Plants

Methods

n/a	Involved in the study	
☒	☐	ChIP-seq
☒	☐	Flow cytometry
☒	☐	MRI-based neuroimaging

Clinical data

Policy information about clinical studies

All manuscripts should comply with the ICMJE guidelines for publication of clinical research and a completed CONSORT checklist must be included with all submissions.

Clinical trial registration: *Provide the trial registration number from ClinicalTrials.gov or an equivalent agency.*

Study protocol: *Note where the full trial protocol can be accessed OR if not available, explain why.*

Data collection: *Describe the settings and locales of data collection, noting the time periods of recruitment and data collection.*

Outcomes: *Describe how you pre-defined primary and secondary outcome measures and how you assessed these measures.*

Plants

Seed stocks: *Report on the source of all seed stocks or other plant material used. If applicable, state the seed stock centre and catalogue number. If plant specimens were collected from the field, describe the collection location, date and sampling procedures.*

Novel plant genotypes: *Describe the methods by which all novel plant genotypes were produced. This includes those generated by transgenic approaches, gene editing, chemical/radiation-based mutagenesis and hybridization. For transgenic lines, describe the transformation method, the number of independent lines analyzed and the generation upon which experiments were performed. For gene-edited lines, describe the editor used, the endogenous sequence targeted for editing, the targeting guide RNA sequence (if applicable) and how the editor was applied.*

Authentication: *Describe any authentication procedures for each seed stock used or novel genotype generated. Describe any experiments used to assess the effect of a mutation and, where applicable, how potential secondary effects (e.g. second site T-DNA insertions, mosiacism, off-target gene editing) were examined.*
